# The basic domain of Suv39h2 buffers mitoxantrone-induced heterochromatin destabilization

**DOI:** 10.1016/j.isci.2026.115626

**Published:** 2026-04-08

**Authors:** Kalina M. Świst-Rosowska, Reagan W. Ching, Birgit Koschorz, Carmen Galan, Bettina Engist, Thomas Jenuwein

**Affiliations:** 1 Department of Epigenetics, Max Planck Institute of Immunobiology and Epigenetics (MPI-IE), Stübeweg 51, 79108 Freiburg, Germany; 2 Gold Standard Diagnostics Madrid, Calle de los Hermanos Garcia Noblejas 39, 28037 Madrid, Spain

**Keywords:** epigenetics

## Abstract

Suv39h1 and Suv39h2 are core components of mouse heterochromatin, where they direct H3K9me3, which is recognized by HP1. In mouse embryonic fibroblasts, heterochromatin retention modes of Suv39h enzymes differ from HP1 and are not sensitive to compounds that impair liquid-liquid phase separation. Suv39h2 contains an N-terminal basic domain that is also present in around 23% of annotated Suv39h orthologs. The Suv39h2 basic domain provides resistance to chromatin-destabilizing agents, such as mitoxantrone and curaxin, and protects H3K9me3 heterochromatin from unfolding or chemically induced histone eviction. This protective function of the basic domain can be transferred to Suv39h1 as an N-terminal fusion. Together, these findings identify the Suv39h2 basic domain as a structural component of heterochromatin and suggest that basic domain extensions help to buffer heterochromatin destabilization.

## Highlights

• Mitoxantrone is a potent compound to disrupt heterochromatin integrity
• The basic domain of Suv39h2 protects heterochromatin from destabilizing compounds
• The protective function of the basic domain transfers to Suv39h1 via N-terminal fusion
• The basic domain of Suv39h2 is a structural component of heterochromatin

## Graphical abstract

**Figure:**
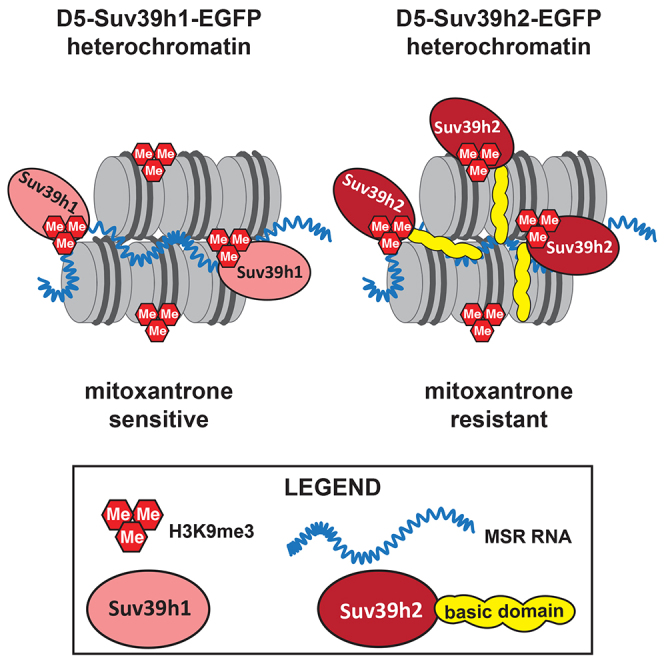

## Introduction

Heterochromatin is one of the two ground states of eukaryotic chromatin^1^ and has important functions in protecting genome integrity and in stabilizing gene expression programs.^2^^,^^3^ In mouse cells, a biochemical pathway has been described in which the core heterochromatic enzymes, the Suv39h lysine methyltransferases (KMT), direct H3K9me3, which is then recognized by heterochromatin protein 1 (HP1).^1^

Mouse pericentric heterochromatin is organized into large repetitive arrays^4^ containing >10,000 copies of a 234 bp, AT-rich sequence, the major satellite repeat (MSR).^5^ A considerable fraction of MSR sequences maintains transcriptional competence,^6^^,^^7^ and it has been proposed that chromatin-associated MSR RNA can form an RNA-nucleosome scaffold at mouse heterochromatin.^8^ RNA can provide an additional affinity for Suv39h enzymes,^8^^,^^9^^,^^10^ and HP1 association with heterochromatin is long known to be sensitive to RNaseA digestion.^11^^,^^12^

There are two Suv39h enzymes, which deposit H3K9me3 at mouse pericentric heterochromatin: Suv39h1^13^ and Suv39h2.^14^ While both enzymes share similar chromodomains and SET domains, Suv3h2 has an extended N-terminal domain, which is enriched in positively charged amino acids and therefore called the basic domain (BD).^8^^,^^14^ While both enzymes are expressed throughout mouse embryonic development, Suv39h2 is selectively found in testes, where it remains chromatin-bound during male meiosis.^14^

Recent biophysical advances have indicated that heterochromatin organization is further modulated by liquid-liquid phase separation (LLPS)^15^^,^^16^ or resembles polymer globules.^17^^,^^18^ HP1α contains an intrinsically disordered region (IDR) that imparts the ability to phase separate and condense chromatin *in vitro.*^19^ Other heterochromatin components with an IDR, such as MeCP2^20^ or linker histone H1,^21^ were also proposed to facilitate heterochromatin compartmentalization via LLPS. While these biophysical models help to explain the dynamic organization of heterochromatin, studies comparing heterochromatin structure between different mouse species have indicated that the underlying sequence of the satellite DNA repeats largely dictates MeCP2 localization^22^ and the higher-order packaging of heterochromatin.^23^

In this study, we address whether Suv39h1^13^ or Suv39h2^14^ enzymes directly participate in the structural stability of mouse heterochromatin. We generated mouse embryonic fibroblast (MEF) cell lines expressing EGFP-tagged Suv39h1 or EGFP-tagged Suv39h2 in a *Suv39h double-null* background, referred to as D5.^24^^,^^25^ With this system, we reveal that Suv39h2 is more stably associated with heterochromatin than Suv39h1. This heterochromatin association is not disrupted by reagents that break liquid-to-liquid phase transitions, such as 1,6-hexanediol; however, it is sensitive to chromatin destabilizing agents, such as mitoxantrone and curaxin (cbl-0137). We show that the BD of Suv39h2 provides resistance to these compounds by protecting heterochromatin from unfolding and histone eviction. This property of the Suv39h2 BD can be transferred to Suv39h1 as an N-terminal fusion. Together, these data reveal that the BD of Suv39h2 contributes to heterochromatin integrity by buffering heterochromatin destabilization.

## Results

### A basic domain N-terminal extension is present in a subset of Suv39h enzymes

**Figure 1:**
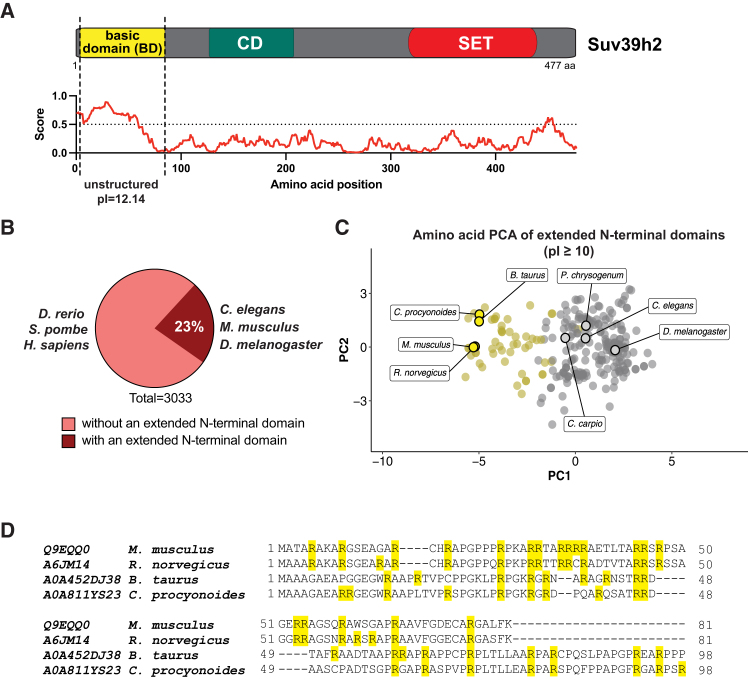
A basic domain N-terminal extension is present in a subset of Suv39h enzymes (A) Schematic representation of the domains in mouse Suv39h2 enzyme (477 aa). The basic domain (BD) is represented in yellow, the chromodomain (CD) in turquoise, and the SET domain in red. The IUPred2 algorithm predicts the basic domain to be intrinsically disordered. Also indicated is the isoelectric point of the basic domain (pI = 12.14). (B) Pie chart representing the percentage of Suv39h enzymes with extended N-terminal domains, as they were filtered from all annotated Suv39h protein orthologs in the UniProt database (total of 3033 Suv39h protein orthologs). 23% of annotated Suv39h protein entries contain an extended N-terminal domain (indicated by the dark-red segment), including Suv39h enzymes from *C. elegans*, *M. musculus,* and *D. melanogaster*. (C) Principal component analysis (PCA) of amino acid composition of extended N-terminal domains of Suv39h enzymes that have a pI equal to or higher than 10. Mouse Suv39h2-BD similar domains are presented in yellow, and mouse Suv39h2-BD distinct domains in gray. Representative organisms from both clusters are indicated with a black overlay. (D) Alignment of amino acid sequences of basic domains of Suv39h enzymes from *M. musculus*, *R. norvegicus*, *B. taurus,* and *C. procyonides*. Arginine residues are highlighted in yellow.

The mouse genome contains two copies of *Suv39h* genes, which encode for Suv39h1 (412 aa)^13^ or Suv39h2 (477 aa).^14^ Suv39h1 and Suv39h2 share the evolutionarily conserved chromo and SET domains, but Suv39h2 also has an additional N-terminal domain that is enriched in positively charged amino acids (primarily arginines). This BD is 81 aa long and has an isoelectric point (pI) of 12.1. Computational analysis predicts that the BD is an IDR of Suv39h2 (Figure 1A).

Human SUV39H2 does not contain a BD. We therefore asked how common it is for Suv39h enzymes to have an extended N-terminal domain. We extracted all available Suv39h protein sequences from the UniProt database^26^ and filtered those with N-terminal extensions beyond the N-terminus of Suv39h1 and being larger than 20 aa (see STAR Methods). This analysis revealed that among the more than 3,000 Suv39h protein entries, 23% contain an extended N-terminal domain. While set-25 of *Caenorhabditis elegans*, SU(VAR)3–9 of *Drosophila melanogaster,* and Suv39h2 of *Mus musculus* contain an N-terminal BD, it is absent in Suv39h enzymes from *Schizosaccharomyces pombe*, *Danio rerio,* and *Homo sapiens* (Figure 1B).

We also found that around 40% of these N-terminal domains have a pI equal to or higher than 10. Principal component analysis of the amino acid composition of these positively charged domains (see STAR Methods) further clustered them into mouse Suv39h2-BD similar (yellow) or mouse Suv39h2-BD distinct (gray) (Figure 1C). The BD of Suv39h enzymes from other rodents (e.g., *Rattus norvegicus*), hoofed animals (e.g., *Bos taurus*), and the raccoon dog (*Canis procyonoides*) are very similar to the mouse Suv39h2 BD, while those present in Suv39h enzymes from some fungi (e.g., *Penicillium chrysogenum*), nematodes (e.g., *C. elegans*), insects (e.g., *D. melanogaster*), and fishes (e.g., *Cyprinus carpio*) are dissimilar. Although the amino acid sequences of representative Suv39h2-BD similar domains are not highly conserved, they are all heavily enriched in arginines (Figure 1D). Further, while amino acid sequences of extended N-terminal domains of Suv39h orthologs among phylogenetically distant organisms are also not conserved, they consistently display an enrichment in basic residues and a high isoelectric point, suggesting a conserved physicochemical property. Together, this analysis shows that basic N-terminal domains are present in many Suv39h orthologs.

### Suv39h2 is more stably associated with heterochromatin than Suv39h1

To address functional differences between Suv39h1 and Suv39h2, we generated stable cell lines expressing either Suv39h1-EGFP or Suv39h2-EGFP in *Suv39h double-null* (D5) immortalized MEFs (see STAR Methods) (Figure S1A). Pericentric heterochromatin can be visualized as round DAPI-dense foci in MEF cells. D5 MEF cells stably expressing Suv39h1-EGFP (D5-Suv39h1-EGFP) or Suv39h2-EGFP (D5-Suv39h2-EGFP) were FACS-sorted, and comparable expression levels were confirmed by western blot (Figure S1B). Suv39h1-EGFP and Suv39h2-EGFP did restore global levels of H3K9me3 in D5 MEF cells similar to those found in wild-type (W8) MEF cells (Figure S1B). Immunofluorescence microscopy further confirmed that Suv39h1-EGFP and Suv39h2-EGFP localized to DAPI-dense foci and reestablished pericentric H3K9me3 and focal enrichment of HP1α (Figure S1C).

**Figure 2:**
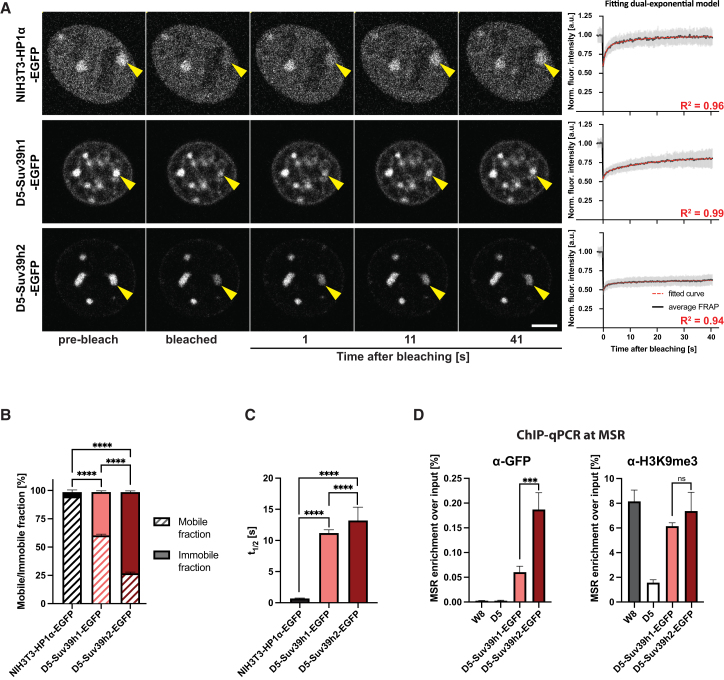
Suv39h2 is more stably associated with heterochromatin than Suv39h1 (A) Representative FRAP images of NIH3T3-HP1α-EGFP, D5-Suv39h1-EGFP, and D5-Suv39h2-EGFP MEF cells. The images show pre-bleach, bleach, and 1, 11, 41 s after-bleach conditions. Bleached EGFP-foci are indicated with a yellow arrow. Scale bar is 5 μm. Average FRAP curves fitted to a dual exponential model are presented in the right. For each condition, *n* ≥ 30 EGFP-foci were bleached. (B) Bar graph quantifying mobile (dashed) and immobile (solid) fractions of EGFP-tagged proteins in NIH3T3-HP1α-EGFP (black), D5-Suv39h1-EGFP (light-red), and D5-Suv39h2-EGFP (dark-red) MEF cells. Values were calculated from the fitted curves of the FRAP data. Asterisks indicate statistically significant differences (*p* < 0.0001, ∗∗∗∗, Tukey test). Error bars represent the standard error of the fitted parameters obtained from the nonlinear regression of averaged FRAP recovery curves. (C) Bar graph for half-maximal recovery time after FRAP of EGFP-tagged proteins in NIH3T3-HP1α-EGFP (black), D5-Suv39h1-EGFP (light-red), and D5-Suv39h2-EGFP (dark-red) MEF cells. Values were calculated from the fitted curves of the FRAP data. Asterisks indicate statistically significant differences (*p* < 0.0001, ∗∗∗∗, Tukey test). Error bars represent the standard error of the fitted parameters obtained from the nonlinear regression of averaged FRAP recovery curves. (D) ChIP-qPCR to detect the enrichment of Suv39h1-EGFP (light-red) and Suv39h2-EGFP (dark-red) (left) or of H3K9me3 (right) at MSR heterochromatin in wild-type (W8), *Suv39h double-null* (D5), D5-Suv39h1-EGFP, and D5-Suv39h2-EGFP MEF cells. Data are shown as mean ± SD from *n* = 3 independent biological replicates. Asterisks indicate statistically significant differences (*p* = 0.0002,∗∗∗, ns is not significant, Tukey test).

To investigate the heterochromatin association of Suv39h enzymes, we performed fluorescence recovery after photo bleaching (FRAP). As a control, we used an immortalized mouse fibroblast cell line—NIH3T3 MEF cells—expressing HP1α-EGFP^27^ (see STAR Methods). FRAP data were normalized and fitted to a dual-exponential model, and the parameters of binding kinetics were calculated (Figure 2A). From the recovery curve of HP1α-EGFP, we calculated 97% mobile and 3% immobile fractions (Figure 2B). These data are in agreement with previous reports showing that HP1α is a highly mobile protein that dynamically interacts with heterochromatin.^28^^,^^29^ For Suv39h1-EGFP, we detect a 60% mobile fraction and a 40% immobile fraction. For Suv39h2-EGFP, only 22% of the protein is found in the mobile fraction and 78% in the immobile fraction. This indicates that the majority of Suv39h2-EGFP remains stably associated with heterochromatin (Figure 2B). We then calculated the half-maximal recovery time (t_1/2_) to gauge the kinetics of Suv39h enzymes interacting with heterochromatin. The t_1/2_ value for HP1α-EGFP of 0.7 s is consistent with it being a highly dynamic protein. The half-maximal recovery times for Suv39h1-EGFP (11.3 s) and Suv39h2-EGFP (13.2 s) were considerably longer, showing that both enzymes require more time to associate with heterochromatin (Figure 2C).

We then performed ChIP-qPCR to investigate the relative enrichment of Suv39h1-EGFP and Suv39h2-EGFP at MSR heterochromatin. Suv39h2-EGFP is approximately 4-fold more enriched at MSR DNA than Suv39h1-EGFP (Figure 2D, left). Despite this difference, both Suv39h1-EGFP and Suv39h2-EGFP can restore the levels of H3K9me3 similar to those observed in W8 MEF cells (Figure 2D, right). Together, the data indicate that Suv39h2-EGFP is a highly immobile protein with robust retention at pericentric heterochromatin.

### Suv39h1 and Suv39h2 do not participate in liquid-to-liquid phase transitions

Proteins with IDR are prone to undergo liquid-to-liquid phase separation (LLPS) when their concentration reaches a critical range.^30^ HP1α has an IDR, and it has been proposed that the biophysical property of HP1α to phase separate can contribute to heterochromatin formation.^15^^,^^16^^,^^31^ To examine whether Suv39h2, where the BD is intrinsically disordered, can undergo LLPS *in vivo*, we incubated D5-Suv39h1-EGFP and D5-Suv39h2-EGFP MEF cells with increasing concentrations of 1,6-hexanediol - a reagent described to interfere with phase separation by disrupting weak hydrophobic interactions.^32^ While we observed rapid dispersion of HP1α from DAPI-dense foci at 2.5%–10% 1,6-hexanediol, both Suv39h1-EGFP and Suv39h2-EGFP persist at pericentric heterochromatin, even at 10% 1,6-hexanediol (Figure S2). It is of note that a concentration of 10% 1,6-hexanediol (which is often used in LLPS experiments) is toxic to MEF cells by impairing their nuclear membrane integrity,^32^ which results in a decrease of nuclear volume (Figure S2). We conclude that, in contrast to HP1α, pericentric localization of Suv39h1-EGFP and Suv39h2-EGFP is not sensitive to 1,6-hexanediol and that their association with heterochromatin is largely not promoted by weak hydrophobic interactions.

### Heterochromatin retention of Suv39h2 is less responsive to RNaseA incubation as compared to HP1α

It is long known that the association of HP1α with DAPI-dense foci is dissolved upon the RNaseA incubation of permeabilized cells.^11^ More recent work has shown that chromatin-associated RNA can facilitate the retention of Suv39h enzymes to heterochromatin.^8^^,^^9^^,^^10^ In particular, the BD of Suv39h2 can bind single-stranded MSR RNA *in vitro.*^8^ To examine whether the more stable association of Suv39h2 with heterochromatin is mediated, at least in part, by RNA, we incubated agarose-immobilized and permeabilized D5-Suv39h1-EGFP and D5-Suv39h2-EGFP MEF cells with increasing concentrations of RNaseA. As a control for the efficiency of RNaseA digestion, we also probed for nucleophosmin 1 (NPM1)—a nucleolar ribonucleoprotein whose localization depends on binding to RNA.^33^ Results from immunofluorescence confocal microscopy show the loss of heterochromatic signal for HP1α upon incubation with RNaseA (25 U) in both D5-Suv39h1-EGFP and D5-Suv39h2-EGFP MEF cells (Figure S3A). By contrast, while the signal for Suv39h1-EGFP over DAPI-dense regions was reduced, Suv39h2-EGFP signals appeared unaltered, as quantified by relative mean fluorescence intensities (Figure S3B). These results indicate that the heterochromatic retention of HP1α, and to a lesser degree of Suv39h1-EGFP, but not of Suv39h2-EGFP, is sensitive to RNaseA digestion. Possibly, the RNA-binding property of the BD of Suv39h2^8^ could insulate MSR RNA in D5-Suv39h2-EGFP MEF cells and protect it from digestion by RNaseA.

### Heterochromatin association of Suv39h2 is more resistant to mitoxantrone exposure than Suv39h1 or HP1α

**Figure 3:**
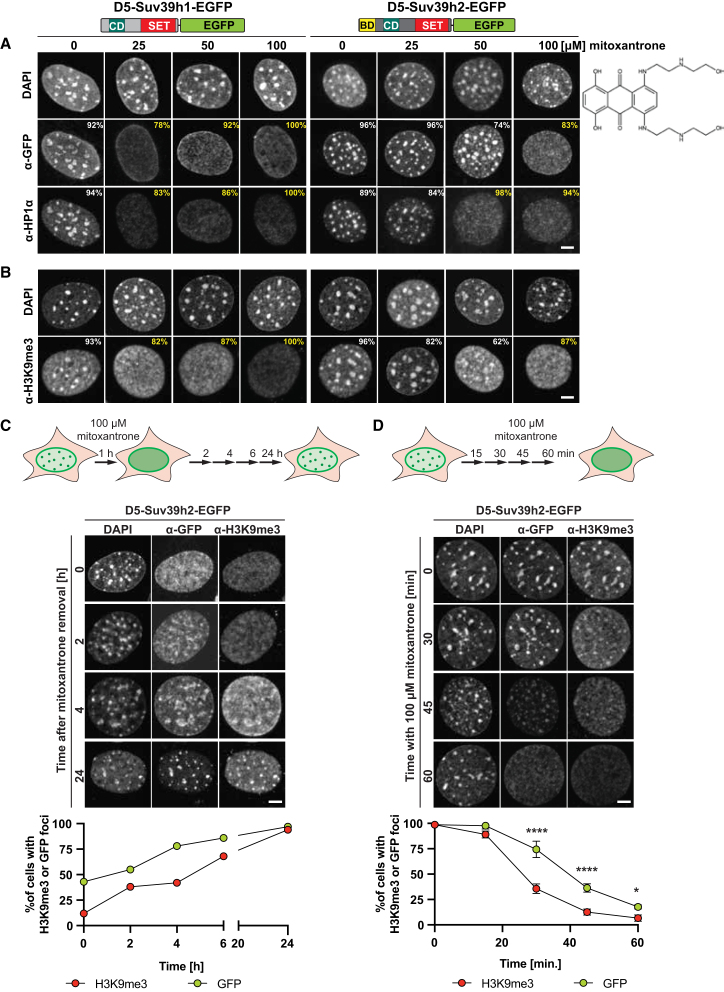
Heterochromatin association of Suv39h2 is more resistant to mitoxantrone exposure than Suv39h1 or HP1α (A) Double-labeling immunofluorescence for Suv39h1-EGFP (left), Suv39h2-EGFP (right), and HP1α in D5-Suv39h1-EGFP and D5-Suv39h2-EGFP MEF cells exposed to a 1 h incubation with 0, 25, 50, and 100 μM mitoxantrone. Cells were labeled with α-GFP and α-HP1α antibodies and counterstained with DAPI. The percentages of cells with focal (white) or dispersed (yellow) fluorescence signals are indicated on the images. For each cell line and condition (i.e., mitoxantrone concentration), *n* ≥ 50 cells were analyzed. Scale bar is 5 μm. The chemical structure of mitoxantrone is shown on the right. (B) Immunofluorescence for H3K9me3 in D5-Suv39h1-EGFP and D5-Suv39h2-EGFP MEF cells exposed to mitoxantrone, as described in (A). (C) Mitoxantrone-mediated dispersion of Suv39h2-EGFP and H3K9me3 is reversible. D5-Suv39h2-EGFP MEF cells were incubated with mitoxantrone for 1 h and then cultivated in mitoxantrone-free medium. Samples were collected 2, 4, 6 (not shown), and 24 h after mitoxantrone removal and double-labeled for GFP and H3K9me3 and counterstained with DAPI. Scale bar is 5 μm. Quantification of the imaging data is shown in the line graph below. For each time point, *n* ≥ 60 cells were analyzed. (D) Time course for the mitoxantrone-mediated dispersion of Suv39h2-EGFP and H3K9me3. D5-Suv39h2-EGFP MEF cells were incubated with 100 μM mitoxantrone for 0, 30, 45, and 60 min. Cells were double-labeled for GFP and H3K9me3 and counterstained with DAPI. Scale bar is 5 μm. Quantification of the imaging data is shown in the line graph below. For each time point, *n* ≥ 65 cells were analyzed (3 independent experiments). Data are shown as mean ± SD. Asterisks indicate statistically significant differences (*p* = 0.0001,∗∗∗, Šidak test).

Since neither 1,6-hexanediol nor RNaseA treatment significantly displaced Suv39h2-EGFP from heterochromatin, we next used mitoxantrone, a small-molecule compound described to dissolve protein-RNA condensates.^34^^,^^35^ We incubated D5-Suv39h1-EGFP and D5-Suv39h2-EGFP MEF cells with increasing concentrations of mitoxantrone for 1 h and then processed them for immunofluorescence confocal microscopy. In D5-Suv39h1-EGFP MEF cells, exposure to 25 μM mitoxantrone results in a loss of heterochromatic enrichment of Suv39h1-EGFP in 78% of analyzed cells and is concomitant with the dispersion of HP1α from DAPI-dense foci (83%). Higher concentrations of mitoxantrone (50 μM and 100 μM) resulted in an increasing fraction of cells (up to 100%) displaying dispersed Suv39h1-EGFP and HP1α signals (Figure 3A, left). Intriguingly, 25 μM mitoxantrone did not delocalize Suv39h2-EGFP or HP1α from heterochromatin. Also, at 50 μM mitoxantrone, Suv39h2-EGFP remained heterochromatin-enriched in 74% of analyzed cells, but HP1α was dispersed (98%). Only mitoxantrone concentrations of 100 μM induced delocalization of Suv39h2-EGFP from DAPI-dense foci in 83% of analyzed cells (Figure 3A, right).

We also examined H3K9me3 in D5-Suv39h1-EGFP and D5-Suv39h2-EGFP MEF cells upon mitoxantrone exposure. H3K9me3 signals are already dispersed at 25 μM mitoxantrone in D5-Suv39h1-EGFP MEF cells (82% of analyzed cells). By contrast, only 100 μM mitoxantrone in D5-Suv39h2-EGFP MEF cells resulted in nearly full (87%) dispersion of H3K9me3 (Figure 3B). Together, these results indicate that the heterochromatic localization of Suv39h2-EGFP is more resistant to mitoxantrone exposure than Suv39h1-EGFP. In addition, mitoxantrone appears to uncouple HP1α dispersion from the heterochromatic retention of Suv39h2-EGFP and H3K9me3.

### Dispersion of H3K9me3 precedes loss of Suv39h enzymes from heterochromatin

We examined whether the mitoxantrone-mediated dispersion of Suv39h2-EGFP and H3K9me3 is reversible. We exposed D5-Suv39h2-EGFP cells with 100 μM mitoxantrone for 1 h and then cultivated the cells in mitoxantrone-free medium. Samples were collected 2, 4, 6, and 24 h post-mitoxantrone incubation and processed for double-labeling immunofluorescence. Both Suv39h2-EGFP and H3K9me3 enrichment at DAPI-dense foci was restored between 6 and 24 h after mitoxantrone removal (Figure 3C, upper). Quantification of the imaging data indicates that at 4 h post-mitoxantrone incubation, around 80% of analyzed cells display enrichment at DAPI-dense regions for Suv39h2-EGFP, while only 40% of those cells had focal H3K9me3 signals (Figure 3C, lower). This is consistent with the restoration of H3K9me3 following the enrichment of Suv39h2-EGFP at DAPI-dense foci. We also note that a 1 h incubation with 100 μM mitoxantrone does not appear to be toxic to the MEF cells.

We next performed a time course experiment to address how fast mitoxantrone disperses Suv39h2-EGFP and H3K9me3 from heterochromatin. We incubated D5-Suv39h2-EGFP MEF cells with 100 μM mitoxantrone for 0, 30, 45, and 60 min and processed them for double-labeling immunofluorescence microscopy. Over this time course, both Suv39h2-EGFP and H3K9me3 became dispersed, with the H3K9me3 signal lost from DAPI-dense foci prior to the dispersion of Suv39h2-EGFP (Figure 3D, upper). Quantification of the imaging data indicates that after 30 min of incubation with mitoxantrone, less than 40% of analyzed cells had a focal H3K9me3 signal compared to 75% of those cells still maintaining heterochromatic localization of Suv39h2-EGFP (Figure 3D, lower). Loss of the H3K9me3 signal from DAPI-dense foci could be a result of active demethylation by the KDM4 family of H3K9-specific demethylases.^36^ However, western blot analysis for H3K9me3 after 1 h of mitoxantrone treatment shows that global H3K9me3 levels remain unchanged (Figure S4). Alternatively, loss of H3K9me3 could be a consequence of mitoxantrone-mediated histone eviction.^37^ Evicted histones are extracted by permeabilization during the preparation of samples for immunofluorescence microscopy; however, they would be present in whole cell extracts used for western blot analyses. The heterochromatin association of Suv39h2-EGFP even after H3K9me3 dispersion is consistent with multivalent interactions of Suv39h2 that comprise binding to MSR RNA,^8^^,^^9^^,^^10^ histone H1,^38^ and atypical Zn-finger factors.^39^

### Suv39h2 buffers histone dispersion against high salt concentrations

The planar structure of mitoxantrone allows for the intercalation of double-stranded DNA and binding to both the major and minor grooves.^40^ In addition to dispersing RNA-protein condensates,^34^^,^^35^ mitoxantrone is an inhibitor of topoisomerase II.^41^ To examine whether topoisomerase inhibition is the cause of the dispersion of HP1α or Suv39h enzymes from heterochromatin, we used etoposide, a topoisomerase II poison,^42^ which does not intercalate DNA.^43^ We incubated D5-Suv39h1-EGFP and D5-Suv39h2-EGFP MEF cells with increasing concentrations of etoposide for 24 h. While etoposide did induce DNA damage, as observed by increasing γH2A.X signals (Figure S5A), it did not delocalize Suv39h1-EGFP, Suv39h2-EGFP, or HP1α from DAPI-dense regions (Figure S5B). We note that a 1 h incubation with mitoxantrone did not activate MSR transcription nor induce higher levels of MSR transcripts (data not shown). The transcriptional activation of MSR transcripts by topoisomerase poisoning is not a fast response, but requires a 24 h incubation with etoposide or genistein (another topoisomerase II inhibitor) to induce the massive upregulation of MSR transcripts without dispersing H3K9me3 or HP1α.^44^

**Figure 4:**
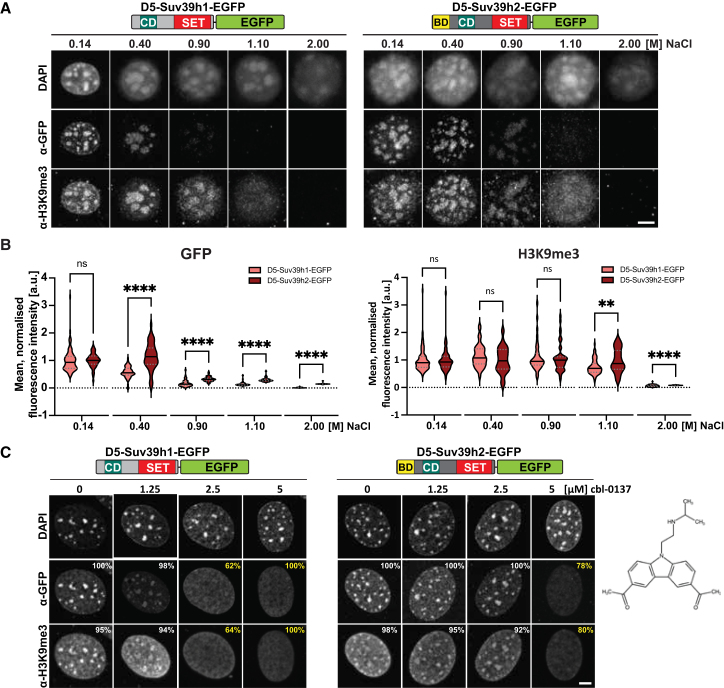
Suv39h2 buffers heterochromatin integrity (A) Double-labeling immunofluorescence for Suv39h1-EGFP (left), Suv39h2-EGFP (right), and H3K9me3 in D5-Suv39h1-EGFP and D5-Suv39h2-EGFP MEF cells that were agarose-embedded, permeabilized, and incubated with 0.14, 0.4, 0.9, 1.1, and 2.0 M NaCl. Cells were labeled with α-GFP and α-H3K9me3 antibodies and counterstained with DAPI. Scale bar is 5 μm. (B) Violin plots show the quantification of mean fluorescence intensities of α-GFP (left) and α-H3K9me3 (right) signals. For each cell line and condition (i.e., NaCl concentration), *n* ≥ 30 cells were analyzed. Median values are indicated. Asterisks indicate statistically significant differences (*p* = 0.0021, ∗∗, *p* < 0.00001, ∗∗∗∗, Mann-Whitney test). (C) Double-labeling immunofluorescence for Suv39h1-EGFP (left), Suv39h2-EGFP (right), and H3K9me3 in D5-Suv39h1-EGFP and D5-Suv39h2-EGFP MEF cells incubated with 0, 1.25, 2.5, and 5 μM cbl-0137. Cells were labeled with α-GFP and αH3K9me3 antibodies and counterstained with DAPI. Percentages of cells with focal (white) or dispersed (yellow) fluorescence signals are indicated on the images. For each cell line and condition (i.e., cbl-0137 concentration), *n* ≥ 60 cells were analyzed. Scale bar is 5 μm. The chemical structure of cbl-0137 is shown on the right.

Mitoxantrone has been described to induce nucleosome unfolding and histone eviction.^37^ To assay for nucleosome unfolding, we used high NaCl concentrations that result in the extraction of histones from chromatin.^45^^,^^46^ Histone modifications have been used as proxies to examine nucleosome stability in previous studies.^47^^,^^48^ Following a published approach,^47^ we immobilized D5-Suv39h1-EGFP and D5-Suv39h2-EGFP MEF cells in agarose. Cells were then permeabilized, incubated with increasing concentrations (0.14, 0.40, 0.90, 1.10, and 2.00 M) of NaCl, and processed for double-labeling immunofluorescence (see STAR Methods). At 0.14 M NaCl, both Suv39h1-EGFP and Suv39h2-EGFP maintained focal enrichment over DAPI-dense foci. At 0.40 M NaCl, Suv39h1-EGFP signals appeared reduced, and at higher NaCl concentrations, Suv39h1-EGFP signals were progressively lost (Figure 4A, left). Intriguingly, the enrichment of Suv39h2-EGFP at DAPI-dense foci and their focal organization remained largely intact at 0.40 M NaCl. Only at 0.90 M NaCl did we begin to observe reduced and dispersed signals for Suv39h2-EGFP. At higher NaCl concentrations, the signal for Suv39h2-EGFP was lost (Figure 4A, right). Co-staining for H3K9me3 indicated a pattern that mirrored the localization of Suv39h1-EGFP and Suv39h2-EGFP. However, H3K9me3 signals remained at higher levels, as compared to Suv39h1-EGFP and Suv39h2-EGFP levels, until NaCl concentrations reached 1.10 M (Figure 4A). The quantification of the mean fluorescence intensities confirmed that Suv39h2-EGFP has a greater potential than Suv39h1-EGFP to buffer histone dispersion as a result of high salt (starting at 0.40 M NaCl). Also, D5-Suv39h2-EGFP MEF cells can maintain higher levels of H3K9me3 at 1.10 M NaCl as compared to D5-Suv39h1-EGFP (Figure 4B). In permeabilized cells, active processes such as transcription, histone modification, and chromatin remodeling are probably not fully active because cofactors and metabolites are extracted. Thus, the progressive loss of the H3K9me3 signal with increasing NaCl concentrations is likely to indicate that histones are evicted from chromatin.

### Suv39h2 protects heterochromatin from histone eviction

High salt concentrations can attenuate not only electrostatic interactions between nucleosomes and DNA, but also disrupt protein-protein and protein-chromatin association. To minimize these confounding effects, we used curaxin to specifically examine chromatin destabilization and histone eviction. Curaxins are small-molecule compounds that inhibit the histone chaperone facilitates chromatin transcription (FACT), and induce nucleosome unfolding and histone eviction.^37^^,^^49^ We incubated D5-Suv39h1-EGFP and D5-Suv39h2-EGFP MEF cells with increasing concentrations (1.25, 2.5, and 5 μM) of the curaxin cbl-0137 for 1 h. While Suv39h1-EGFP and H3K9me3 start to disperse from DAPI-dense foci at 2.5 μM cbl-0137 (62–64% of analyzed cells), Suv39h2-EGFP and H3K9me3 signals remain enriched at heterochromatin. Only at 5 μM cbl-0137 were the signals for Suv39h1-EGFP lost, or for Suv39h2-EGFP significantly decreased (Figure 4C). Interestingly, HP1α begins to delocalize from heterochromatin at 1.25 μM cbl-0137 in D5-Suv39h1-EGFP MEF cells (31% of analyzed cells), but still co-localizes with DAPI-dense foci and Suv39h2-EGFP signals at 2.5 μM cbl-0137 in D5-Suv39h2-EGFP MEF cells (55% of analyzed cells) (Figure S6A). These data indicate that cbl-0137 can delocalize Suv39h enzymes and HP1α from heterochromatin and disperse H3K9me3. They also show that D5-Suv39h2-EGFP MEF cells are more resistant to cbl-0137-mediated heterochromatin disruption and histone eviction than D5-Suv39h1-EGFP MEF cells.

To further confirm a protective function for Suv39h2 in buffering cbl-0137-induced nucleosome unfolding, we performed micrococcal (MNase) digestion (see STAR Methods) of nuclei from D5-Suv39h1-EGFP and D5-Suv39h2-EGFP MEF cells that were either untreated or treated with 2.5 or 5.0 μM cbl-0137. The incubation of untreated samples from D5-Suv39h1-EGFP and D5-Suv39h2-EGFP MEF cells with increasing amounts of MNase reveals a regularly spaced nucleosome ladder that is best resolved at digestion with 24 U of MNase (Figure S6B and quantification in Figure S6C). Upon incubation with 2.5 μM cbl-0137, this nucleosome ladder was still present in samples from D5-Suv39h2-EGFP MEF cells, but was nearly lost in samples from D5-Suv39h1-EGFP MEF cells. Incubation with 5 μM cbl-0137 resulted in the absence of a nucleosome ladder in both D5-Suv39h1-EGFP and D5-Suv39h2-EGFP MEF cell samples. The progressive loss of a nucleosome ladder with increasing concentrations of cbl-0137 reflects nucleosome destabilization and histone eviction. Although analyzed at a global scale and merely inferred to include H3K9me3 nucleosomes, the data support the interpretation that Suv39h2-EGFP, more than Suv39h1-EGFP, protects heterochromatin from curaxin-induced destabilization.

### The protective function of the basic domain can be transferred to Suv39h1 as an N-terminal fusion

With the aim to directly demonstrate a protective function of the BD, we generated two additional MEF cell lines expressing either a Suv39h2 mutant lacking the BD—D5-Suv39h2ΔBD-EGFP (BD mutant) or an N-terminal BD fusion with Suv39h1—D5-BD-Suv39h1-EGFP (BD fusion) (Figure S7A). Both cell lines express comparable levels of EGFP-tagged Suv39h enzymes (Figure S7B), which localize to DAPI-dense foci and restore H3K9me3 and HP1α at pericentric heterochromatin (Figure S7C).

**Figure 5:**
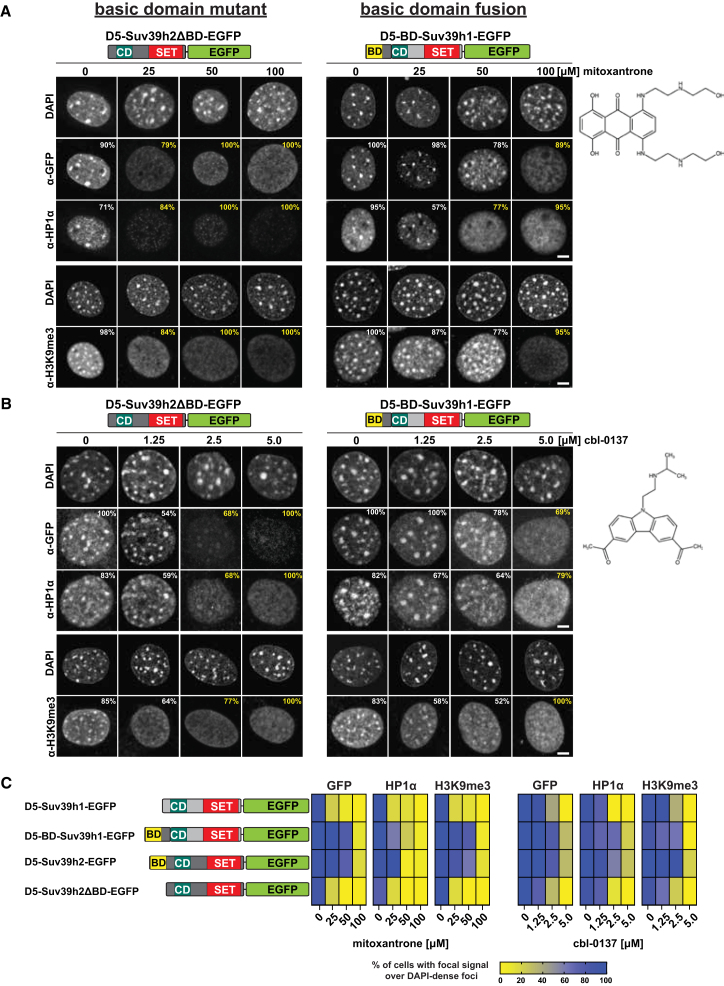
The protective function of the basic domain can be transferred to Suv39h1 as an N-terminal fusion (A and B) Immunofluorescence of D5-Suv39h2ΔBD-EGFP (left) and D5-BD-Suv39h1-EGFP (right) MEF cells incubated with 0, 25, 50, and 100 μM mitoxantrone (A) or 0, 1.25, 2.5, and 5 μM cbl-0137 (B). Cells were double-labeled with α-GFP and α-HP1α or single-labeled with α-H3K9me3 antibodies and counterstained with DAPI. The percentages of cells with focal (white) or dispersed (yellow) fluorescence signals are indicated on the images. For each cell line and condition (i.e., mitoxantrone or cbl-0137 concentration), *n* ≥ 50 cells were analyzed. Scale bars are 5 μm. The chemical structures of mitoxantrone and cbl-0137 are shown on the right. (C) Heatmap shows the quantification of imaging data for GFP, HP1α, and H3K9me3 localization in D5-Suv39h1-EGFP, D5-BD-Suv39h1-EGFP, D5-Suv39h2-EGFP, and D5-Suv39h2ΔBD-EGFP MEF cells exposed to increasing concentrations of either mitoxantrone or cbl-0137. This heatmap summarizes the imaging analyses from Figures 3A, 3B, 4C, 5A, 5B, and S6A. Percentages of cells with fluorescence signals over DAPI-dense foci are indicated by yellow (no overlap, dispersed) to blue (overlap, focal enrichment) gradient.

The deletion of the BD weakened the resistance of Suv39h2ΔBD-EGFP to both mitoxantrone (Figure 5A, left) and cbl-0137 (Figure 5B, left) mediated dispersion from heterochromatin. In contrast to full-length Suv39h2-EGFP, which required 100 μM mitoxantrone for dispersion (Figure 3A), a mitoxantrone concentration of 25 μM was sufficient to displace Suv39h2ΔBD-EGFP (79% of analyzed cells) and HP1α (84% of analyzed cells) from DAPI-dense foci and also to disperse H3K9me3 (Figure 5A, left). Similarly, concentrations of 1.25 and 2.5 μM cbl-0137 were sufficient to delocalize Suv39h2ΔBD-EGFP (46%–68% of analyzed cells) and HP1α (41%–68% of analyzed cells) from DAPI-dense foci, with H3K9me3 signals (36%–77% of analyzed cells) also being dispersed (Figure 5B, left). Concentrations above 2.5 μM cbl-0137 were required to delocalize full-length Suv39h2-EGFP from heterochromatin (Figure 4D).

The fusion of the BD to Suv39h1-EGFP strengthened the resistance of BD-Suv39h1-EGFP to both mitoxantrone (Figure 5A, right) and curaxin cbl-0137 (Figure 5B, right) mediated dispersion from heterochromatin. In contrast to Suv39h1-EGFP, which required as little as 25 μM mitoxantrone for dispersion (Figure 3A), a mitoxantrone concentration above 50 μM was required to fully displace BD-Suv39h1-EGFP from heterochromatin. While HP1α was broadly dispersed at 50 μM mitoxantrone (77% of analyzed cells), the majority of signals for BD-Suv39h1-EGFP (78% of analyzed cells) and H3K9me3 (77% of analyzed cells) still overlapped with DAPI-dense foci (Figure 5A, right). Heterochromatin association of BD-Suv39h1-EGFP was also more resistant (as compared to Suv39h1-EGFP, see Figure 4C, left) to higher concentrations of cbl-0137, and only at 5.0 μM cbl-0137 did BD-Suv39h1-EGFP (69% of analyzed cells) and HP1α (79% of analyzed cells) become delocalized from heterochromatin, with H3K9me3 signals (100% of analyzed cells) being fully dispersed (Figure 5B, right).

We compared the results from Figures 5A and 5B to the results from Figures 3A, 4C, and S6A provides a comparative analysis of D5 MEF cells rescued with Suv39h1-EGFP, Suv39h2-EGFP, Suv39h2ΔBD-EGFP, and BD-Suv39h1-EGFP. As shown in the heatmap (Figure 5C), Suv39h enzymes containing the BD (i.e., Suv39h2-EGFP and BD-Suv39h1-EGFP) are more resistant to mitoxantrone or cbl-0137-mediated displacement from heterochromatin and, to a large degree, preserve focal HP1α and H3K9me3 localization. Since both mitoxantrone and cbl-0137 are nucleosome destabilizing compounds, we would like to propose that the BD of Suv39h2 provides a buffering function in stabilizing a nucleosome scaffold at heterochromatin. This property of the Suv39h2 BD can be transferred to Suv39h1 as an N-terminal fusion.

## Discussion

### Different retention modes of Suv39h enzymes and HP1α at heterochromatin

Heterochromatin association of core heterochromatin proteins often depends on multivalent interactions^50^ that include binding to H3K9me3, linker histone H1,^38^ contacts with chromatin-associated satellite RNA,^8^^,^^9^^,^^10^ and formation of liquid-like condensates.^15^^,^^16^^,^^31^ HP1α is more dynamic than Suv39h1 and Suv39h2 at heterochromatin, as observed from the FRAP analyses. Suv39h2 is most stably associated with heterochromatin, in agreement with a previous report.^51^ Notably, Suv39h1 and Suv39h2 are not dispersed by 1,6-hexanediol, a compound that breaks weak hydrophobic interactions and delocalizes HP1α from heterochromatin. These data specify that Suv39h1 and Suv39h2 do not participate in LLPS. Mitoxantrone disperses HP1α at concentrations where Suv39h2 remains enriched at DAPI-dense foci. This could relate to mitoxantrone dissolving RNA-protein condensates.^34^ Also, HP1α is rapidly delocalized from heterochromatin upon RNaseA incubation, while Suv39h2, and to a lesser degree Suv39h1, persist at DAPI-dense foci. Together, these data indicate that heterochromatin retention modes for HP1α are largely guided by RNA interaction and dynamic participation in LLPS, as reported in previous studies.^11^^,^^15^^,^^16^ Heterochromatin retention modes for Suv39h enzymes appear to be different.

HP1α and Suv39h enzymes also contain the evolutionarily conserved chromodomain, which binds to H3K9me3.^52^ We are not excluding the chromodomain in contributing to the different residence times between Suv39h1 and Suv39h2 as observed in the FRAP studies, or the greater enrichment at heterochromatin of Suv39h2 observed in ChIP. Chromodomains interact with methylated lysines via an aromatic cage composed of hydrophobic amino acids.^53^^,^^54^ Compared to Suv39h1, Suv39h2 has two tryptophan residues within its aromatic cage, whereas Suv39h1 has only one tryptophan residue. This additional tryptophan increases the cation-π potential^55^ and therefore could strengthen the binding to H3K9me3.

In our previous work, we analyzed isolated nuclei and purified polynucleosomes with RNaseA, which led us to propose an RNA-nucleosome scaffold model for mouse heterochromatin.^8^ In the current work, we used permeabilized MEF cells, which present a more native heterochromatin context and preserve multivalent interactions. While the heterochromatin association of Suv39h2 in permeabilized D5-Suv39h2-EGFP cells appears less sensitive to RNaseA digestion, the BD of Suv39h2 could insulate RNA and protect an RNA-nucleosome scaffold, consistent with our previous model.

### The basic domain of Suv39h2 as a structural component of heterochromatin

An important result from our study is that the BD of Suv39h2 participates in the protection of pericentric heterochromatin against chromatin destabilizing agents such as high salt (electrostatic interaction), mitoxantrone (DNA intercalation and RNA-protein condensates), and curaxin (histone eviction).

The BD of Suv39h2 is predicted to be unstructured and composed of 25% arginine residues (pI = 12.1). Arginines preferably bind to narrow minor grooves of the DNA double helix.^56^ It has been shown recently that a high density of A/T-base pairs reshapes MSR DNA to contain a specifically narrowed minor groove.^23^ For example, Hmga1, another heterochromatin component, binds to AT-rich sequences in *M. musculus* MSR repeat DNA and organizes heterochromatin into the classic DAPI-dense heterochromatic foci.^23^^,^^57^^,^^58^ Hmga1 has a basic pI (pI = 10.3) and is known to undergo a disordered-structured transition upon binding to the minor groove of DNA via its arginine-rich AT-hook domains.^59^ The BD of Suv39h2 shares some sequence similarity with one of the AT-hooks of Hmga1 (Figure S8). Although currently not resolved, it is tempting to infer that the BD of Suv39h2 could directly interact with narrowed minor grooves in AT-rich MSR repeat DNA. We previously reported that the BD of Suv39h2 does not bind to dsDNA.^8^ However, for these *in vitro* DNA binding experiments, the DNA oligonucleotides were only 35 bp long and could only form fewer than four minor grooves. In addition to being AT-rich, MSR DNA presents a high density of poly(dA:dT) tracts.^23^ Poly(dA:dT) tracts not only narrow the minor groove of DNA,^56^ but they also cause local distortion of DNA^60^ and disfavor nucleosome formation.^61^ One MSR unit (234 bp) contains around 20 poly(dA:dT) tracts of at least four dA or dT nucleotides.^23^ If there is direct binding of the BD of Suv39h2 to MSR DNA, then this could neutralize the nucleosome destabilizing property of poly(dA:dT) tracts and protect a nucleosome scaffold at mouse heterochromatin.

Similar to the BD of Suv39h2, linker histone H1.4 also has a highly basic pI (pI = 11.1) and an intrinsically disordered C-terminal domain (“basic region”) that is involved in DNA interaction and chromatin condensation.^62^^,^^63^^,^^64^ The BD of Suv39h2 shares only very modest sequence similarity to the “basic region” in linker histone H1.4 (Figure S8). While our data suggest that the BD of Suv39h2, such as Hmga1^23^^,^^57^^,^^58^ or linker histone H1,^38^ participates in the structure of mouse heterochromatin, future experiments are required to directly examine its DNA interaction or nucleosome scaffolding properties.

Although we have analyzed the function of the BD of Suv39h2 in MEF cells, Suv39h2 is highly expressed in testes.^14^ During spermatogenesis, chromatin undergoes extensive remodeling, including the replacement of most histones with protamines.^65^ Protamine 1 (PRM1) and protamine 2 (PRM2) are arginine-rich proteins with a high isoelectric point (pI > 12) that are expressed in late spermatids to compact the paternal genome into a transcriptionally inert state.^65^ Intriguingly, PRM2 (107 aa) shares some sequence similarity across stretches of arginine residues with the BD of Suv39h2 and is also largely unstructured (Figure S8). It is thus not unconceivable that the BD of Suv39h2 may be particularly relevant to help transmit Suv39h2-mediated H3K9me3 signals during spermatogenesis.^66^^,^^67^

While this notion needs to be explored in future experiments, our work identifies the BD of Suv39h2 as a structural component of heterochromatin with a function in buffering heterochromatin destabilization.

### Limitations of the study

While this study provides previously undescribed insights into the role of the BD of Suv39h2 in protecting the stability of pericentric heterochromatin in MEF cells, several limitations should be acknowledged.

First, our work does not reveal the detailed molecular mechanism for the protective function of the BD of Suv39h2. Although we show that the BD of Suv39h2 protects heterochromatin integrity from exposure to various chromatin destabilizing compounds, the underlying biochemical interactions remain to be elucidated.

Second, while NaCl, mitoxantrone, and cbl-0137 globally disrupt chromatin structure, our analysis was centered on H3K9me3 heterochromatin and DAPI-dense heterochromatic foci. This was largely guided by IF localization for H3K9me3, Suv39h enzymes, and HP1α, and therefore, our conclusions are restricted to this chromatin compartment.

Third, although the robust chromatin association of Suv39h2 and its dependence on the BD suggest that the BD may directly protect nucleosomes from mitoxantrone- and/or cbl-0137-induced histone eviction, we cannot exclude the potential involvement of other heterochromatin factors (e.g., Hmga1,^27^^,^^57^^,^^59^ linker histone H1^38^, Suv4-20h,^68^ MeCP2,^22^ atypical Zn-F factors^39^), histone chaperones (e.g., DAXX^69^ and FACT^37^^,^^49^) or chromatin remodelers (e.g., ATRX^70^). However, as H3K9me3 turnover is known to be very slow,^71^^,^^72^ such indirect effects are likely limited within the experimental time frame of 1 h.

Lastly, the strength of our study resides in the *in situ* and imaging-based analysis of mouse heterochromatin. Testing the direct involvement of the BD of Suv39h2 in nucleosome stability *in vitro* would be informative; however, simplified *in vitro* systems would not provide an unambiguous test of the phenomenon described in this study. In particular, minimal nucleosome-based assays lack key architectural and regulatory features of pericentric heterochromatin, including repetitive MSR DNA, MSR-derived RNA, H3K9me3-modified nucleosomes, HP1α, linker histone H1, and additional targeting factors. In addition, some chromatin-destabilizing agents used in this study act through mechanisms that require additional chromatin-associated factors (for example, curaxins acting through the FACT complex), limiting their applicability in minimal *in vitro* systems. Consequently, negative results in such systems would not exclude the possibility that the BD acts directly on heterochromatin *in vivo*, as simplified assays cannot reliably distinguish between the absence of a direct stabilizing effect and the absence of essential heterochromatin context. Dissecting these mechanisms using biochemical reconstitution represents an important future direction beyond the scope of the present study, which focuses on heterochromatin stabilization in its native cellular context.

## Resource availability

### Lead contact

Further information and requests for resources and reagents should be directed to the lead contact, Kalina Świst-Rosowska (swist@ie-freiburg.mpg.de).

### Materials availability

All unique reagents used in this study are available from the lead contact with a completed materials transfer agreement.

### Data and code availability

Original western blot images have been deposited at Mendeley at [DOI: https://doi.org/10.17632/wccscb4vt3.1] and are publicly available as of the date of publication. Microscopy data reported in this paper will be shared upon request by the lead contact. This paper analyzes publicly available data from Uniprot. Any additional information required to reanalyze the data reported in this paper is available from the lead contact upon request.

## Acknowledgments

We thank Tony Hyman (MPI Dresden) for pointing out mitoxantrone as a more potent reagent than 1,6-hexanediol in breaking RNA-protein condensates. We are grateful to Petra Kindle, Visnja Jevtic, Roland Pohlmeyer, and Sergiy Avilov from the Imaging facility at the MPI-IE for help with image acquisition and analysis.

Research in the laboratory of T.J. is supported by the 10.13039/501100004189Max Planck Society.

## Author contributions

K.S.R, R.W.C., and B.K. conducted the experiments. B.K., C.G., and B.E. generated the expression constructs and the cell lines. K.S.R. and R.W.C. analyzed the data. K.S.R. and T.J. wrote the manuscript. T.J. supervised the project.

## Declaration of interests

The authors declare no competing interests.

## STAR★Methods

### Key resources table

**Table:** 

REAGENT or RESOURCE	SOURCE	IDENTIFIER
**Antibodies**		

goat polyclonal α-GFP	Rockland	Cat#: 600-141-215; RRID: AB_1961516
rabbit polyclonal α-GFP	Invitrogen	Cat#: A11122; RRID: AB_221569
mouse monoclonal α-GAPDH	SantaCruz Biotechnology	Cat#: sc-32233; RRID: AB_627679
rabbit monoclonal α-HP1α	Abcam	Cat#: ab109028; RRID: AB_10858495
rabbit polyclonal α-H3K9me3	Abcam	Cat#: 8898; RRID: AB_306848
mouse polyclonal α-H2A.X (Ser139)	Millipore	Cat#: 05-636; RRID: AB_309864
rabbit monoclonal α-H3	Abcam	Cat#: ab176842; RRID: AB_2493104
rabbit polyclonal α-H3K9me3	Jenuwein lab	# 1926
mouse monoclonal α-Nucleophosmin	Abcam	Cat#: ab10530; RRID: AB_297271

**Chemicals, peptides, and recombinant proteins**		

High-glucose Dulbecco's Modified Eagle Medium (DMEM)	Sigma-Aldrich	Cat#: D6171
HyClone Characterized Fetal bovine serum	Cytiva	Cat#: SH30071.03
L-glutamine	Sigma-Aldrich	Cat#: G7513
β-mercaptoethanol	Gibco	Cat#: 31350-010
MEM Non-essential Amino Acid Solution (100×)	Sigma-Aldrich	Cat#: M7145
sodium pyruvate solution	Sigma-Aldrich	Cat#: S8636
penicillin-streptomycin	Sigma-Aldrich	Cat#: P4333
puromycin dihydrochloride	Sigma-Aldrich	Cat#: P9620
XhoI restriction enzyme	New England Biolabs	Cat#: R0146S
AflII restriction enzyme	New England Biolabs	Cat#: R05020S
PvuI restriction enzyme	New England Biolabs	Cat#: R3150S
XFect Transfection reagent	Takara	Cat#: 631317
DPBS	Gibco	Cat#: 14190-094
RIPA	Pierce	Cat#: 89900
cOmplete EDTA-free protease inhibitors	Roche	Cat#: 11873580001
paraformaldehyde	Santa Cruz	Cat#: sc-281692
triton X-100	Sigma-Aldrich	Cat#: X100
VECTASHIELD Antifade Mounting Medium with DAPI	Vector Leboratories	Cat#: H-1200
donkey serum	Jackson ImmunoResearch	Cat#: 017-000-121
phenol red-free DMEM	Gibco	Cat#: 21063029
1,6-hexanediol	Sigma-Aldrich	Cat#: 240117
low melting point agarose	Bethesda Scientific	Cat#: V3841
RNaseA	Thermo Scientific	Cat#: EN0531
mitoxantrone	Selleckchem	Cat#: S2485
cbl-0137	Selleckchem	Cat#: S0507
formaldehyde	Sigma-Aldrich	Cat#: F8775
glycine	Sigma-Aldrich	Cat#: G8898
SDS	Sigma-Aldrich	Cat#: L3771
EDTA	Sigma-Aldrich	Cat#: 798681
tris-HCl	Sigma-Aldrich	Cat#: 108315
NaCl	Sigma-Aldrich	Cat#: S5886
dynabeads Protein G for Immunoprecipitation	Invitrogen	Cat#: 10003D
LiCl	Merck	Cat#: 310468
sodium deoxycholate	Sigma-Aldrich	Cat#: D6750
NaHCO_3_	Merck	Cat#: S6297
proteinase K	Thermo Scientific	Cat#: EO0491
MNase	Thermo Scientific	Cat#: EN0181
SYBR Select Master Mix	Applied Biosystems	Cat#: 4472908
SYBR Safe DNA Gel Stain	Invitrogen	Cat#: S33102
HEPES	Merck	Cat#: H3375
MgCl_2_	Merck	Cat#: M8266
CaCl_2_	Merck	Cat#:449709

**Critical commercial assays**		

NucleoSpin Gel and PCR Clean-up Kit	Macherey-Nagel	Cat#: 740609.250

**Deposited data**		

Original western blot images	Mendeley: https://doi.org/10.17632/wccscb4vt3.1	N/A

**Experimental models: Cell lines**		

W8 immortalized mouse embryonic fibroblasts	Jenuwein lab	W8
D5 immortalized mouse embryonic fibroblasts	Jenuwein lab	D5
NIH 3T3 HP1α-EGFP immortalized mouse embryonic fibroblasts	Jenuwein lab	N/A

**Oligonucleotides**		

MSR_for (5’-TGGAATATGGC GAGAAAACTG)	Ching et al.^73^	N/A
MSR_rev (5’-AGGTCCTTCAG TGGGCATTT)	Ching et al.^73^	N/A

**Recombinant DNA**		

pCAGGS-Suv39h1-EGFP-IRES-Puro	Velazquez et al.^8^	Laboratory of Thomas Jenuwein Database: #693
pCAGGS-Suv39h2-EGFP-IRES-Puro	Velazquez et al.^8^	Laboratory of Thomas Jenuwein Database: #694
pCAGGS-Suv39h2ΔBD-EGFP-IRES-Puro	Velazquez et al.^8^	Laboratory of Thomas Jenuwein Database: #703
pCAGGS-BD-Suv39h1-EGFP-IRES-Puro	This study	Laboratory of Thomas Jenuwein Database: #696

**Software and algorithms**		

R (v4.4.3)	R Core Team	https://www.r-project.org/
Clustal Omega	EMBL-EBI	https://www.ebi.ac.uk/Tools/msa/clustalo/
GraphPad Prism 10	GraphPad Software	https://www.graphpad.com/
Peptides (v2.4.6)	Charif & Lobry, 2007	https://cran.r-project.org/package=Peptides
ggplot2 (v3.5.1)	Hadley Wickham	https://cran.r-project.org/package=ggplot2
ggrepel (v0.9.6)	Slowikowski et al.	https://cran.r-project.org/package=ggrepel
IUPred2	Dosztányi et al., 2005	https://iupred2a.elte.hu/
Zen Black software	Zeiss	https://www.zeiss.com/microscopy/int/products/microscope-software/zen.html
Fiji (v2.9.0)	Schindelin et al., 2012	https://fiji.sc/
Marvin JS (22.11.1)	Chemaxon internal	http://www.chemaxon.com

### Experimental model and study participant details

#### Cell lines and culture maintenance

Wild-type (W8) and *Suv39h double-null* (D5) MEF cells were isolated from male mouse embryos of a mixed genetic background of 129/Sv and C57BL/6J origin. D5 MEF cells were generated by homologous recombination as described in Peters et al.^25^ NIH3T3 (male origin) MEF cells expressing the N-terminal fusion of EGFP to HP1α were generated by gene-trap mutagenesis, as reported in Fodor et al.^27^ W8, D5, and NIH3T3-HP1α-EGFP MEF cells were maintained in high-glucose DMEM (Sigma-Aldrich) supplemented with 10% FBS (Cytiva), 2 mM L-glutamine (Sigma-Aldrich), 0.1 mM β-mercaptoethanol (Gibco), 1×non-essential amino acids (Sigma-Aldrich), 1 mM sodium pyruvate (Sigma-Aldrich), 100 U/ml penicillin, and 100 μg/ml streptomycin (Sigma-Aldrich). D5 MEF cells expressing EGFP-tagged Suv39h enzymes were maintained in MEF cell medium supplemented with 10 μM puromycin (Sigma-Aldrich). The cells were cultured in a Heracell 240 CO_2_ incubator (Thermo Scientific) at 37°C with 5% CO_2_. Cell line identity was based on their original derivation and previously published characterization, and no additional authentication was performed. All cell lines were routinely tested for mycoplasma contamination and confirmed to be negative. MEF cells were originally derived from mouse embryos generated in-house as part of previously published studies.

### Method details

#### Identification of Suv39h enzymes with and without an extended N-terminal domain

All protein sequences containing the term “Suv39h” in any of the description fields were retrieved from the UniProt database (accessed on 05 March 2025).^26^ From this initial dataset, using R (version 4.4.3), sequences were filtered to include only those with “histone” in the protein name, while entries containing “prdm” or “setmar” were excluded. This filtering step yielded 3033 Suv39h-related protein sequences. These sequences were aligned using Clustal Omega.^74^^,^^75^ Based on the resulting multiple sequence alignment, we selected sequences whose N-terminal domains extended beyond that of the *Mus musculus* Suv39h1 (UniProt ID: O54864), resulting in a subset of 888 sequences. These were then re-aligned with Clustal Omega. In the second alignment, all sequences were truncated at the position corresponding to amino acid 81 in the *Mus musculus* Suv39h2 sequence (UniProt ID: Q9EQQ0). Sequences longer than 20 amino acids after truncation were retained, yielding a final dataset of 694 sequences with extended N-terminal domains. The data were visualized in a pie chart using GraphPad Prism 10.

#### Analysis of amino acid composition and isoelectric point of N-terminal domains of Suv39h

The amino acid composition of the 694 extended N-terminal domains was calculated using a custom R script, while their isoelectric points (pI) were computed using the Peptides package (version 2.4.6). Sequences with a pI ≥ 10 were selected for further analysis. Principal component analysis (PCA) was then performed based on the amino acid composition of these high-pI sequences. The results were visualized as a dot plot using the ggplot2 (version 3.5.1) and ggrepel (version 0.9.6) packages in R (version 4.4.3).

The alignment of the amino acid sequence of extended N-terminal domains from Suv39h2 of *M. musculus, R. norvegicus, B. taurus,* and *C. procyonoides* was performed using UniProt alignment tool.^26^

#### Generation of the constructs to express EGFP-tagged Suv39h enzymes

Plasmids for overexpression of Suv39h fusion proteins were generated by cloning synthetic gene fragments into the pCAGGS-EGFP-IRES-Puro backbone. Specifically, pCAGGS-Suv39h1-EGFP-IRES-Puro, pCAGGS-Suv39h2-EGFP-IRES-Puro, pCAGGS-Suv39h2ΔBD-EGFP-IRES-Puro, and pCAGGS-BD-Suv39h1-EGFP-IRES-Puro were constructed to express Suv39h1-EGFP, Suv39h2-EGFP, Suv39h2ΔBD-EGFP, and BD-Suv39h1-EGFP fusion proteins, respectively. The synthetic genes encoded full-length Suv39h1 (amino acids 1–412), full-length Suv39h2 (1–477), a basic domain deletion variant Suv39h2ΔBD (deletion of T3–K81), and a fusion of the Suv39h2 N-terminal domain (1–119) with Suv39h1 (44–412). Gene fragments were flanked by XhoI and AflII restriction sites (IDT) and cloned into the backbone using XhoI and AflII restriction enzymes (NEB), as previously described in Velazquez et al.^8^ All plasmid sequences were confirmed by Sanger sequencing.

#### Generation of MEF cells expressing EGFP-tagged Suv39h enzymes

To generate D5-Suv39h1-EGFP, D5-Suv39h2-EGFP, D5-Suv39h2ΔBD-EGFP, and D5-BD-Suv39h1-EGFP cell lines, the plasmids pCAGGS-Suv39h1-EGFP-IRES-Puro, pCAGGS-Suv39h2-EGFP-IRES-Puro, pCAGGS-Suv39h2ΔBD-EGFP-IRES-Puro, and pCAGGS-BD-Suv39h1-EGFP-IRES-Puro were first linearized using the *PvuI* restriction enzyme (NEB). Subsequently, 5 μg of each linearized plasmid was transfected into D5 MEF cells^25^ using the Xfect transfection reagent (Clontech). Twenty-four hours post-transfection, cells were selected with MEF medium supplemented with 10 μM puromycin for 7 days. To enrich for MEF cells expressing EGFP-tagged Suv39h constructs, fluorescence-activated cell sorting (FACS) was performed. Cells were maintained as mixed populations.

#### Western blot

MEF cells were trypsinized, harvested, and washed twice with PBS. To prepare protein lysates, cell pellets were lysed in RIPA buffer (Pierce) containing protease inhibitors (cOmplete EDTA-free protease inhibitors, Roche), and sonicated (30 s ON, 30 s OFF, 15 cycles) (Bioruptor, Diagenode). These samples were then processed for western blotting (10 μg protein lysate per lane) with antibodies against GFP (600-141-215, Rockland; 1:1000), GAPDH (sc-32233, Santa Cruz; 1:1000), HP1α (ab109028, Abcam; 1:1000), H3K9me3 (ab8898, Abcam; 1:1000), *γ*H2A.X (Millipore, 05-636; 1:500), and H3 (ab176842, Abcam; 1:50000).

#### Immunofluorescence

Cells were fixed at room temperature with 4% paraformaldehyde (PFA) for 15 min, followed by permeabilization with 0.5% Triton X-100 for 5 min. After washing, samples were incubated with primary antibodies against GFP (600-101-215, Rockland; 1:1000) and either HP1α (ab109028, Abcam; 1:1000) or H3K9me3 (clone 1926, Laboratory of Thomas Jenuwein; 1:1000). Cells were counterstained with DAPI, and samples were mounted using VECTASHIELD Antifade Mounting Medium with DAPI (Vector Laboratories, H-1200-10).

Imaging was performed at 63×magnification using an LSM780 confocal microscope (Zeiss). Maximum intensity projections were generated using ZEN Black software (Zeiss). Images were processed using identical acquisition settings across conditions.

The percentage of dispersed cells was calculated as the proportion of cells with a fluorescent signal not colocalizing with DAPI-dense foci relative to the total number of cells analyzed. Quantification of imaging data from mitoxantrone- or cbl-0137-treated samples was visualized as a heatmap using GraphPad Prism 10.

#### Fluorescent recovery after photobleaching (FRAP)

Approximately 2 × 10^6^ cells were seeded onto μ-Dish 35 mm high ibiTreat dishes (Ibidi) and incubated overnight at 37 °C in MEF cell medium supplemented with 10 μM puromycin. On the day of the experiment, the medium was replaced with high-glucose, phenol red-free DMEM (Gibco), supplemented with 10% fetal bovine serum (Cytiva), 2 mM L-glutamine, 0.1 mM β-mercaptoethanol, 1×non-essential amino acids, 1 mM sodium pyruvate, and 100 U/ml penicillin/100 μg/ml streptomycin (all from Sigma-Aldrich).

FRAP experiments were performed using a Zeiss LSM780 confocal microscope equipped with a 63×oil-immersion objective and the ZEN Black FRAP module. Image acquisition was conducted at a resolution of 688 × 344 pixels, with a pixel dwell time of 0.76 μs. A circular region of interest (ROI) of 2–5 μm^2^ was selected for bleaching. For each replicate, three ROIs were defined: a bleached ROI (centered on the EGFP focus), a reference ROI (on a separate unbleached EGFP focus), and a background ROI (outside EGFP-labeled regions). Image acquisition consisted of 10 pre-bleach frames, 2 bleach frames, and 188 post-bleach frames, acquired at ∼0.21 s intervals. For each cell line, 30 individual foci were bleached and analyzed.

Data analysis was performed using a custom R script. Fluorescence intensities were normalized using a full (double) normalization approach, and recovery curves were fitted to a dual-exponential model. Mobile and immobile fractions, as well as half-times of recovery (t_1/2_), were calculated as previously described.^76^

#### Chromatin immunoprecipitation (ChIP)-qPCR

ChIP was performed as described previously Montavon et al.,^77^ with minor modifications. Approximately 1×10^7^ trypsinized and washed in FBS-free DMEM MEF cells were resuspended in 4,6 ml FBS-free DMEM and mixed with 312 μl 16% formaldehyde (Sigma-Aldrich). Cells were crosslinked for 10 min at room temperature, and the reaction was quenched by adding glycine to a final concentration of 0.125 M for 5 min. Crosslinked cells were pelleted by centrifugation (5 min, 300×*g*, room temperature) and washed twice with FBS-free DMEM. The pellet was resuspended in 1 ml cold lysis buffer containing 1% SDS (Sigma-Aldrich), 10 mM EDTA (Sigma-Aldrich), 50 mM Tris-HCl (Sigma-Aldrich), pH 8.0), and protease inhibitors (cOmplete EDTA-free, Roche) and lysed for 25 min at 4°C, with rotation. Chromatin was sheared to an average fragment size of 200–800 bp using an S220 Focused-ultrasonicator (Covaris). After sonication, the lysate was cleared by centrifugation (10 min, 16,000×*g*, 4°C) and diluted 10-fold with dilution buffer (1% Triton X-100 (Sigma-Aldrich), 2 mM EDTA (Sigma-Aldrich), 167 mM NaCl (Sigma-Aldrich), 20 mM Tris-HCl (Sigma-Aldrich), pH 8.1) supplemented with protease inhibitors. Chromatin equivalent to 10 μg of DNA was incubated overnight at 4°C with rotation with 4 μg of α-GFP (A11122, Invitrogen) or 4 μg of α-H3K9me3 (ab8898, Abcam) antibody. Following incubation, 20 μl of pre-washed Protein G magnetic beads (Invitrogen) were added, and samples were incubated for 2 h at 4°C with rotation. Beads were washed for 5-10 min at 4°C with rotation in the following sequence: three times with low-salt buffer (1% SDS, 1% Triton X-100, 2 mM EDTA, 150 mM NaCl, 20 mM Tris-HCl, pH 8.1), once with high-salt buffer (1% SDS, 1% Triton X-100, 2 mM EDTA, 500 mM NaCl, 20 mM Tris-HCl, pH 8.1), once with LiCl buffer (0.25 M LiCl (Sigma-Aldrich), 0.5% NP-40 (Sigma-Aldrich), 0.5% sodium deoxycholate (Sigma-Aldrich), 1 mM EDTA, 10 mM Tris-HCl, pH 8.1), and once with TE buffer (10 mM Tris-HCl, pH 8.1, 1 mM EDTA). Chromatin was eluted from beads in 200 μl elution buffer (1% SDS, 100 mM NaHCO_3_ (Sigma-Aldrich)) for 1 h at room temperature with rotation. Crosslinks were reversed by incubation with 1 μl RNaseA (10 mg/ml, Thermo Scientific) for 1 h at 37°C followed by overnight incubation with 2 μl Proteinase K (20 mg/ml, Thermo Scientific) at 65°C. DNA was purified using a NucleoSpin Gel and PCR Clean-up Kit (Macherey-Nagel) and eluted in 105 μl NE buffer (from the kit).

For quantitative PCR, purified ChIP DNA was diluted 100× and 2 μl were mixed with 0.25 μM MSR-specific primers (see key resources table) and 5 μl SYBR Select Master Mix (Applied Biosystems) in a total reaction volume of 10 μl, and amplified using a QuantStudio 6 Flex Real-Time PCR System (Applied Biosystems) under the following conditions: 95°C for 10 min, followed by 40 cycles of 95°C for 15 s and 60°C for 1 min. Enrichment relative to input DNA was calculated using the ΔCt method.^78^

#### 1,6-hexanediol treatment of MEF cells

Approximately 1×10^5^ cells were seeded into each well of a 4-chamber culture slide (Falcon) and incubated overnight at 37 °C in MEF medium supplemented with 10 μg/ml puromycin. The following day, the medium was replaced with fresh MEF medium supplemented with 10 μM puromycin containing 0, 2.5, 5.0, or 10% 1,6-hexanediol (Sigma-Aldrich), and the cells were incubated for 3 min at 37 °C. After treatment, slides were briefly washed with PBS and processed for immunofluorescence staining.

#### RNaseA treatment of MEF cells

RNaseA treatment was performed as described in Ching et al.^73^ Microscope slides (Superfrost, Epredia) were coated with a 0.7% low-melting-point agarose solution prepared in water and allowed to dry completely before cell harvesting. Approximately 1×10^7^ MEF cells were harvested by trypsinization, washed, and resuspended in PBS. The cell suspension was then mixed in a 1:1 ratio with 1.4% low-melting-point agarose prepared in PBS and maintained at 37 °C. A total of 50 μl of the mixture was spotted onto the agarose-coated slides and covered with a 22 × 22 mm coverslip. Slides were placed on a 37 °C thermoblock for 3 min to allow cell sedimentation, then transferred to an ice-cold metal block for 3 min to solidify the agarose. Coverslips were gently removed, and the slides were briefly washed with PBS. Cells were permeabilized with 0.5% Triton X-100 in PBS for 5 min, followed by three 5-min washes with PBS. Slides were then incubated for 1 h at 37 °C with 20 U RNaseA in digestion buffer (20 mM HEPES pH 7.5, 0.1 mM CaCl_2_, 3 mM MgCl_2_, 100 mM KCl). Following RNaseA digestion, slides were washed three times for 5 min with PBS and fixed with 4% paraformaldehyde (PFA) in PBS for 15 min. Subsequently, slides were stained with antibodies against NPM1 (ab10530, Abcam; 1:1000) and HP1α (ab109028, Abcam; 1:1000), then mounted with VECTASHIELD containing DAPI (Vector Laboratories, H-1200-10). Imaging was performed using a Zeiss LSM880 confocal microscope with a 63×objective. Maximum intensity projections were generated using ZEN Black software (Zeiss). Mean fluorescence intensities of GFP and H3K9me3 per cell were quantified using Fiji (v2.9.0). Normalized data were presented as violin plots, and statistical analyses were conducted in GraphPad Prism 10.

#### Mitoxantrone treatment of MEF cells

Approximately 1×10^5^ cells were seeded into each well of a 4-well chamber slide (Falcon) and incubated overnight at 37 °C in MEF cell medium supplemented with 10 μM puromycin. The following day, the medium was replaced with fresh MEF cell medium supplemented with 10 μM puromycin containing 0, 25, 50, or 100 μM mitoxantrone (Sigma-Aldrich), and cells were incubated for 1 h at 37 °C. After treatment, slides were briefly rinsed with PBS and processed for immunofluorescence staining.

For the time-course experiment, cells were treated with 100 μM mitoxantrone for 15, 30, 45, or 60 min at 37 °C, followed by PBS washing and immunostaining.

For the recovery experiment, cells were first incubated with 100 μM mitoxantrone for 1 h at 37 °C. After washing with PBS, cells were cultured in drug-free medium for 1, 2, 4, 6, or 24 h before being processed for immunostaining.

#### NaCl treatment of MEF cells

NaCl extraction of MEF cells was performed as previously described,^47^ with minor modifications. Microscope slides (Superfrost, Epredia) were pre-coated with 0.7% low-melting-point agarose in distilled water and allowed to dry completely before use. Cells were harvested by trypsinization, washed, and resuspended in PBS. The cell suspension was mixed 1:1 with 1.4% low-melting-point agarose in PBS and maintained at 37 °C. A 50 μl aliquot of the cell-agarose mixture was pipetted onto the pre-coated slides and covered with a 22 × 22 mm coverslip. Slides were incubated on a 37 °C thermoblock for 3 min to allow cell sedimentation, followed by a 3-minute incubation on an ice-cold metal block to solidify the agarose. Coverslips were carefully removed. Cells were permeabilized on ice for 10 min using ice-cold 1% Triton X-100 in PBS containing 5 mM EDTA (PBS/EDTA), then washed three times for 3 min each with ice-cold PBS/EDTA. Slides were subsequently incubated at 4 °C for 30 min in PBS/EDTA containing increasing concentrations of NaCl (0.14 M, 0.4 M, 0.9 M, 1.1 M, and 2.0 M). After salt extraction, slides were again washed three times with PBS/EDTA. Cells were then immunostained with antibodies against GFP (600-101-215, Rockland; 1:1000) and H3K9me3 (clone 1926, Laboratory of Thomas Jenuwein; 1:1000), followed by incubation with appropriate secondary antibodies. Samples were post-fixed in 4% PFA at room temperature for 15 min and mounted using VECTASHIELD Antifade Mounting Medium with DAPI (Vector Laboratories, H-1200-10). Imaging was performed at 63× magnification using an LSM780 confocal microscope (Zeiss), and maximum intensity projections were generated using ZEN Black software (Zeiss). Quantification of mean fluorescence intensity per cell for GFP and H3K9me3 was conducted in Fiji (v2.9.0). Data were normalized and visualized as violin plots in GraphPad Prism 10, which was also used for statistical analysis.

#### Cbl-0137 treatment of MEF cells

For immunostaining experiments, approximately 1 × 10^5^ MEF cells were seeded into each well of a 4-chamber culture slide (Falcon) and incubated overnight at 37 °C in MEF medium supplemented with 10 μM puromycin. The following day, the medium was replaced with fresh MEF cell medium supplemented with 10 μM puromycin containing 0, 1.25, 2.5, or 5 μM cbl-0137 (SelleckChemicals), and cells were incubated for 1 h at 37 °C. After treatment, slides were briefly washed with PBS and processed for immunofluorescence.

For the Mnase digestion assay, approximately 10 × 10^6^ cells were seeded into a 20 cm culture dish and incubated overnight at 37 °C in MEF medium supplemented with 10 μM puromycin. The next day, the medium was replaced with fresh MEF cell medium supplemented with 10 μM puromycin containing 0, 2.5, or 5 μM cbl-0137, and cells were incubated for 1 h at 37 °C. Following treatment, cells were harvested by trypsinization and subjected to MNase digestion.

#### MNase-treatment

The PBS-washed cell pellet was resuspended in hypotonic buffer (20 mM HEPES pH 7.5, 20 mM NaCl, 5 mM MgCl_2_, and 0.1% NP-40) to a final concentration of ∼1×10^7^ cells/ml with an 18G needle and incubated on ice for 10 min. Cell nuclei were centrifuged at 500g at 4°C for 5 min, and the pellet was resuspended in Ex100 (20 mM HEPES pH 7.5, 100 mM NaCl, and 0.5 mM MgCl_2_) and incubated on ice for 15 min. The solution was centrifuged at 500g at 4°C for 5 min, and the resuspension and incubation in Ex100 were repeated. After the second Ex100 incubation and centrifugation, the pellet was resuspended in 500 μl of Ex100. Using a Nanodrop-1000 (ThermoFisher), the cell nuclei were diluted to a final concentration of 200 ng/μl of nucleic acids. CaCl_2_ was then added to a final concentration of 2 mM and incubated at 25°C for 10 min. After incubation, the nuclei solution was divided into 100 μl aliquots, MNase (0, 3, 6, 12, 24, 48 U) (ThermoFisher) was added, and incubated at 25°C for 20 min. The reactions were stopped with the addition of EDTA to a final concentration of 10 mM. The MNase-digested chromatin was purified using a PCR clean-up kit (Machery-Nagel). The purified nucleic acids were then run on a 1% agarose gel and post-stained in SYBR Safe (Invitrogen) for 1 h. Images of the gel were obtained using GelDoc Go Imaging System (Bio-Rad). Line scans were used to quantify the nucleosomal ladder using Fiji (v2.9.0) and plotted using GraphPad Prism 10.

#### Visualization of chemical structures

Chemical structures in Figures 3, 4, 5, S2, S5, and S6 were drawn using Marvin (Chemaxon, version 22.11.1).

### Quantification and statistical analyses

Quantification methods are described above. No datapoints were excluded from the reported analyses. Statistical analyses were performed in GraphPad Prism 10 or R. The statistical test used and the definition of statistical significance in each case are included in the figure legends.
