# Supplementary material for: The basic domain of Suv39h2 buffers mitoxantrone-induced heterochromatin destabilization
Source: iScience. 2026 Apr 8;29(5):115626. doi: 10.1016/j.isci.2026.115626 (PMC13127484; doi:10.1016/j.isci.2026.115626)

iScience, Volume *29*

Supplemental information

The basic domain of Suv39h2 buffers mitoxantrone-induced heterochromatin destabilization

Kalina M. Świst-Rosowska, Reagan W. Ching, Birgit Koschorz, Carmen Galan, Bettina Engist, and Thomas Jenuwein

SUPPLEMENTAL FIGURE LEGENDS

Supplemental Figure 1. Expression and localization of EGFP-tagged Suv39h1 and Suv39h2 in *Suv39h double-null* MEF cells

(A) Schematic representation of Suv39h1 and Suv39h2 EGFP-fusion constructs used in this study. The N-terminal basic domain (BD) of Suv39h2 is indicated in yellow, the chromodomain in turquoise, the SET domains in red, and EGFP in green. Protein diagrams are relative in scale.

(B) Western blot showing the expression levels of Suv39h1-EGFP, Suv39h2-EGFP, HP1α and H3K9me3 in wild-type (W8), *Suv39h double-null* (D5), D5-Suv39h1-EGFP, and D5-Suv39h2-EGFP MEF cells. GAPDH and histone H3 staining are used as loading controls.

(C) Immunofluorescence analyses of W8, D5, D5-Suv39h1-EGFP and D5-Suv39h2-EGFP MEF cells for the localization of GFP, HP1α and H3K9me3 signals. Cells were counterstained with DAPI. Scale bar is 5 μm.

Supplemental Figure 2. Heterochromatin association of Suv39h1-EGFP and Suv39h2-EGFP is insensitive to 1,6-hexanediol exposure

Double-labeling immunofluorescence for Suv39h1-EGFP (left panel), Suv39h2-EGFP (right panel) and HP1α in D5-Suv39h1-EGFP and D5-Suv39h2-EGFP MEF cells incubated with 0, 2.5, 5, and 10% 1,6-hexanediol. Cells were labeled with α-GFP and α-HP1α antibodies and counterstained with DAPI. The percentages of cells with focal (white) or dispersed (yellow) fluorescence signals are indicated on the images. For each cell line and condition (i.e. 1,6-hexanediol concentration), n≧50 cells were analyzed. Scale bar is 5 μm. The chemical structure of 1,6-hexanediol is shown on the right.

Supplemental Figure 3. Heterochromatin retention of Suv39h2 is less responsive to RNaseA incubation

(A, B) Immunofluorescence analysis of agarose-embedded and permeabilized D5-Suv39h1-EGFP and D5-Suv39h2-EGFP MEF cells incubated with 0 U or 25 U of RNaseA. Cells were labeled with α-HP1α (A) or double-labeled with α-GFP and α-NPM1 (B) antibodies and counterstained with DAPI. Violin plots quantifying the relative mean fluorescence intensities observed at 0 U (white) or 25 U (orange) of RNaseA incubation are shown in the panels to the right. For each cell line and condition (i.e. 0 U and 25 U of RNaseA incubation), n≥50 cells were analyzed. Median values are indicated. Asterisks indicate statistically significant differences ($p<0.0001$, ****, Kruskal-Wallis test).

Supplemental Figure 4. Mitoxantrone does not alter H3K9me3 levels

Western blot showing the expression levels of Suv39h1-EGFP, Suv39h2-EGFP and H3K9me3 in untreated wild-type (W8), *Suv39h double-null* (D5) and in D5 MEF cells expressing Suv39h1-EGFP or Suv39h2-EGFP, either untreated or treated with mitoxantrone for 1 h. GAPDH and histone H3 staining are used as loading controls.

Supplemental Figure 5. Suv39h1–EGFP and Suv39h2–EGFP maintain heterochromatin association after etoposide treatment

(A) Western blot showing the expression levels of γH2A.X in wild-type W8 MEF cells exposed to 0, 2.5, 0.5, and 1 µM etoposide. Histone H3 staining is used as a loading control.

(B) Double-labeling immunofluorescence for Suv39h1-EGFP (left panel), Suv39h2-EGFP (right panel) and HP1α in D5-Suv39h1-EGFP and D5-Suv39h2-EGFP MEF cells incubated with 0, 2.5, 0.5, and 1 µM etoposide. Cells were labeled with α-GFP and α-HP1α antibodies and counterstained with DAPI. Percentages of cells with focal (white) fluorescence signals are indicated on the images. For each cell line and condition (i.e., etoposide concentration), n≥38 cells were analyzed. Scale bar is 5 µm. The chemical structure of etoposide is shown on the right.

Supplemental Figure 6. Curaxin-induced dispersion of HP1α from heterochromatin

(A) Double-labeling immunofluorescence for Suv39h1-EGFP (left panel), Suv39h2-EGFP (right panel) and HP1α in D5-Suv39h1-EGFP and D5-Suv39h2-EGFP MEF cells incubated with 0, 1.25, 2.5, and 5 μM cbl-0137. Cells were labeled with α-GFP and α-HP1α antibodies and counterstained with DAPI. Percentages of cells with focal (white) or dispersed (yellow) fluorescence signals are indicated on the images. For each cell line and condition (i.e. cbl-0137 concentration), n≥50 cells were analyzed. Scale bar is 5 μm. The chemical structure of cbl-0137 is shown on the right.

(B) Agarose gel showing nucleosome ladders of MNase-digested chromatin from D5-Suv39h1-EGFP and D5-Suv39h2-EGFP MEF cells treated with 0, 2.5, and 5 μM cbl-0137. MNase digestion was titrated with 3, 6, 12, 24, and 48 U of MNase. Digestion with 24 U MNase, which is quantified in (C), is highlighted by red arrows.

(C) Line scans quantifying the relative intensities of nucleosome ladders after digestion with 24 U MNase.

Supplemental Figure 7. Expression and localization of Suv39h2ΔBD-EGFP and BD-Suv39h1-EGFP in *Suv39h double-null* MEF cells

(A) Schematic representation of Suv39h2ΔBD and BD-Suv39h1 EGFP-fusion mutants used in this study. The N-terminal basic domain (BD) of Suv39h2 is indicated in yellow, the chromodomain in turquoise, the SET domains in red and EGFP in green. Protein diagrams are relative in scale.

(B) Western blot showing the expression levels of Suv39h2ΔBD-EGFP, BD-Suv39h1-EGFP, HP1α, and H3K9me3 in wild-type (W8), *Suv39h double-null* (D5), D5-Suv39h2ΔBD-EGFP and D5-BD-Suv39h1-EGFP MEF cells. GAPDH and histone H3 staining are used as loading controls.

(C) Immunofluorescence analyses of W8, D5, D5-Suv39h2ΔBD-EGFP, and D5-BD-Suv39h1-EGFP MEF cells for the localization of GFP, HP1α and H3K9me3 signals. Cells were counterstained with DAPI. Scale bar is 5 μm.

Supplemental Figure 8. Comparison of the basic domain of Suv39h2 with mouse Hmga1, histone H1.4 and protamines

Left panel: schematic representation and sequence alignment of the Suv39h2 basic domain (BD, yellow) with mouse Hmga1, histone H1.4, and protamines PRM1 and PRM2. Hmga1 AT-hooks are shown in blue, the H1.4 globular domain (GD) in orange and the H1.4 „basic region” in green. Protein diagrams are relative in scale, and the total amino acid length is indicated for each protein. In the alignments, identical residues are written in red and highlighted in light gray. Right panel: isoelectric points of mouse Hmga1, histone H1.4, PRM1 and PRM2. and predicted intrinsically disordered regions, as determined using the IUPred2 algorithm.

Supplemental figure 1

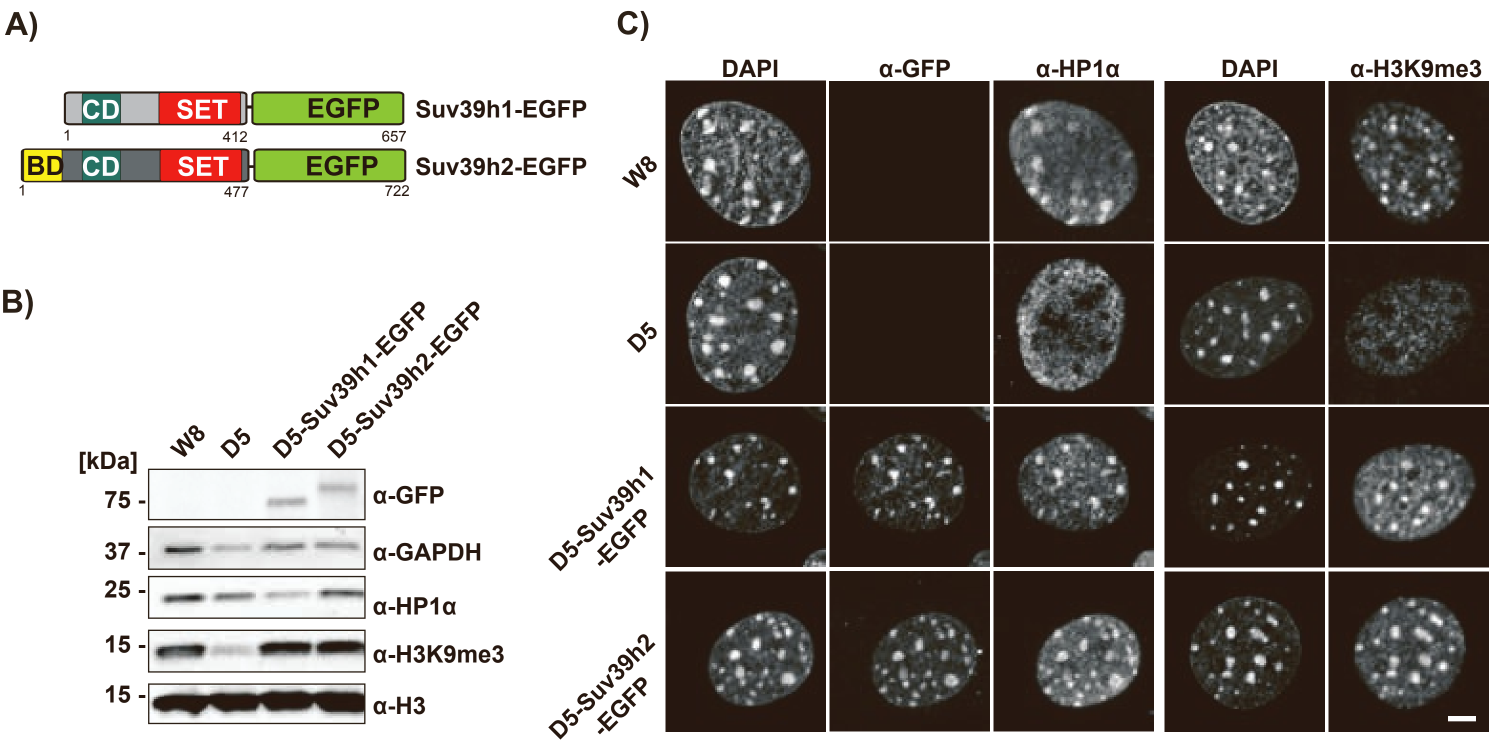

Supplemental figure 2

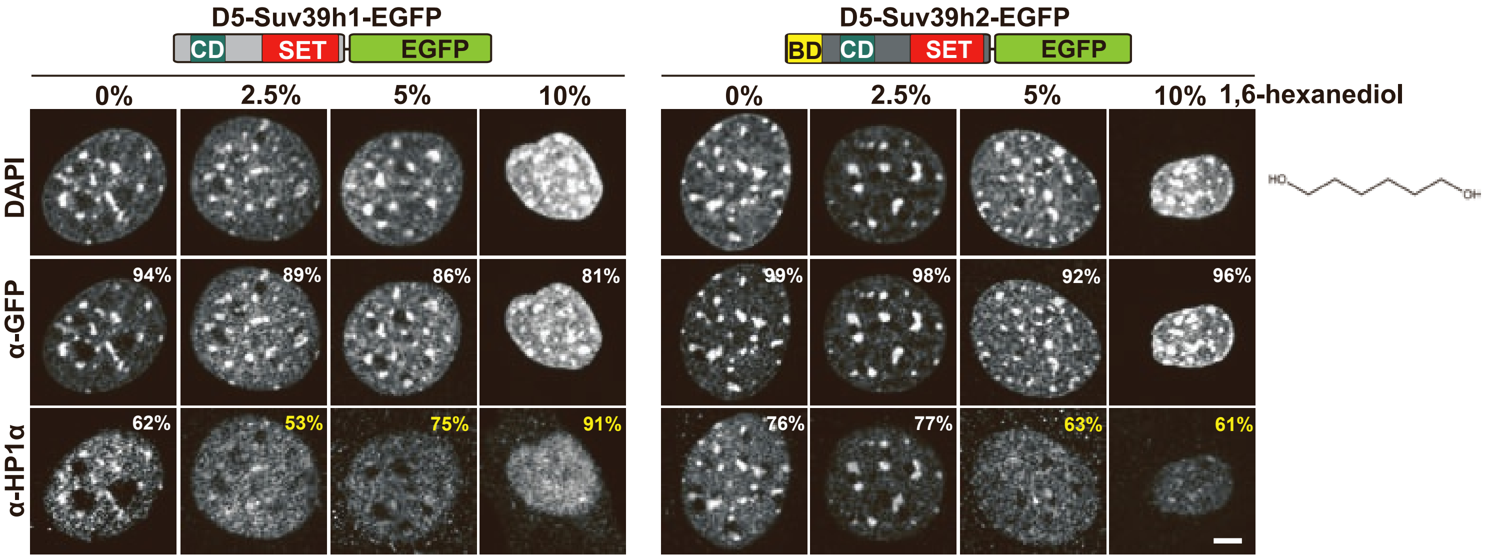

Supplemental figure 3

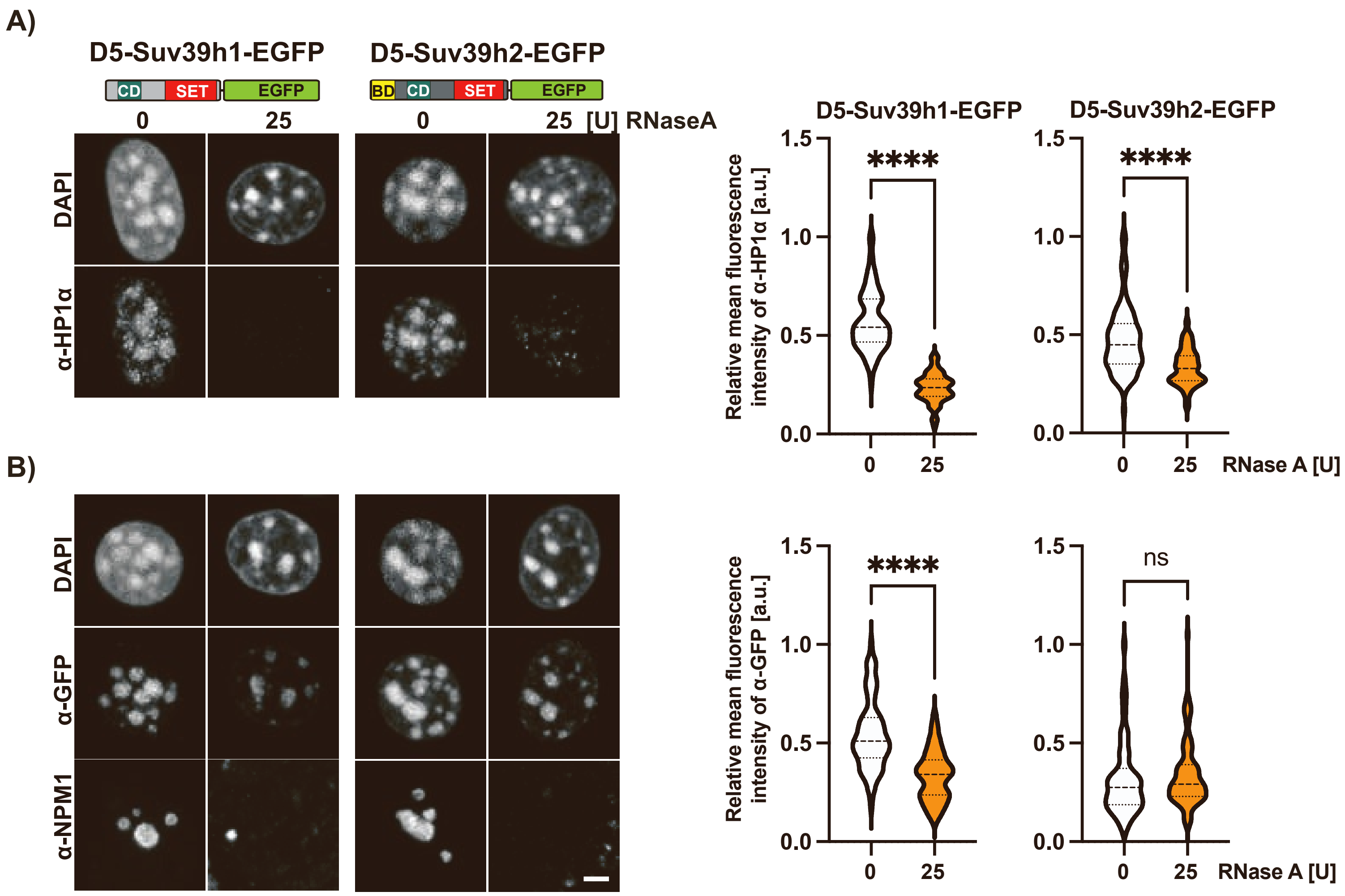

Supplemental figure 4

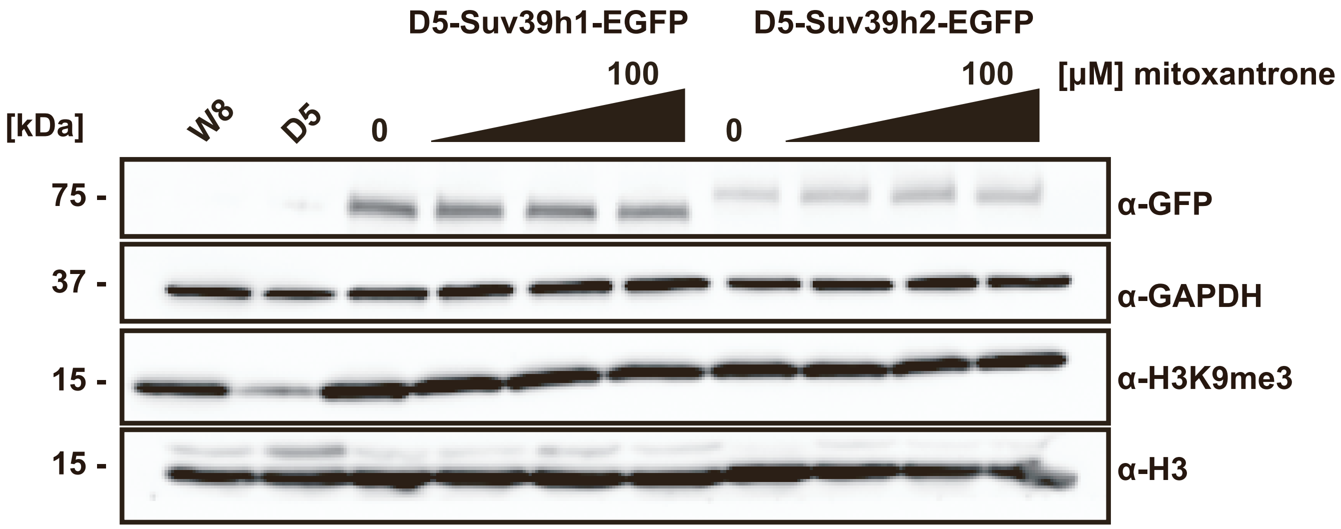

Supplemental figure 5

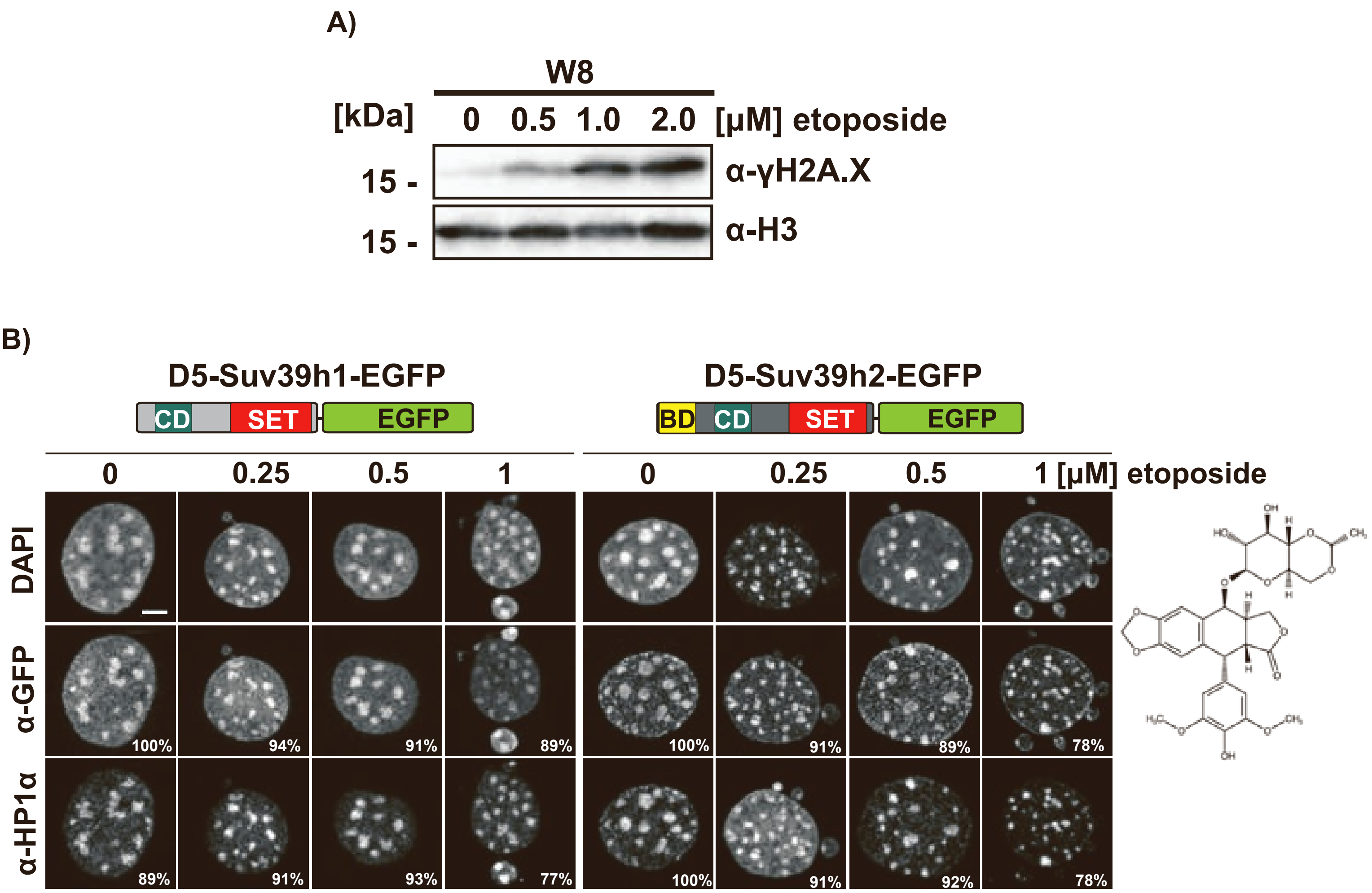

Supplemental figure 6

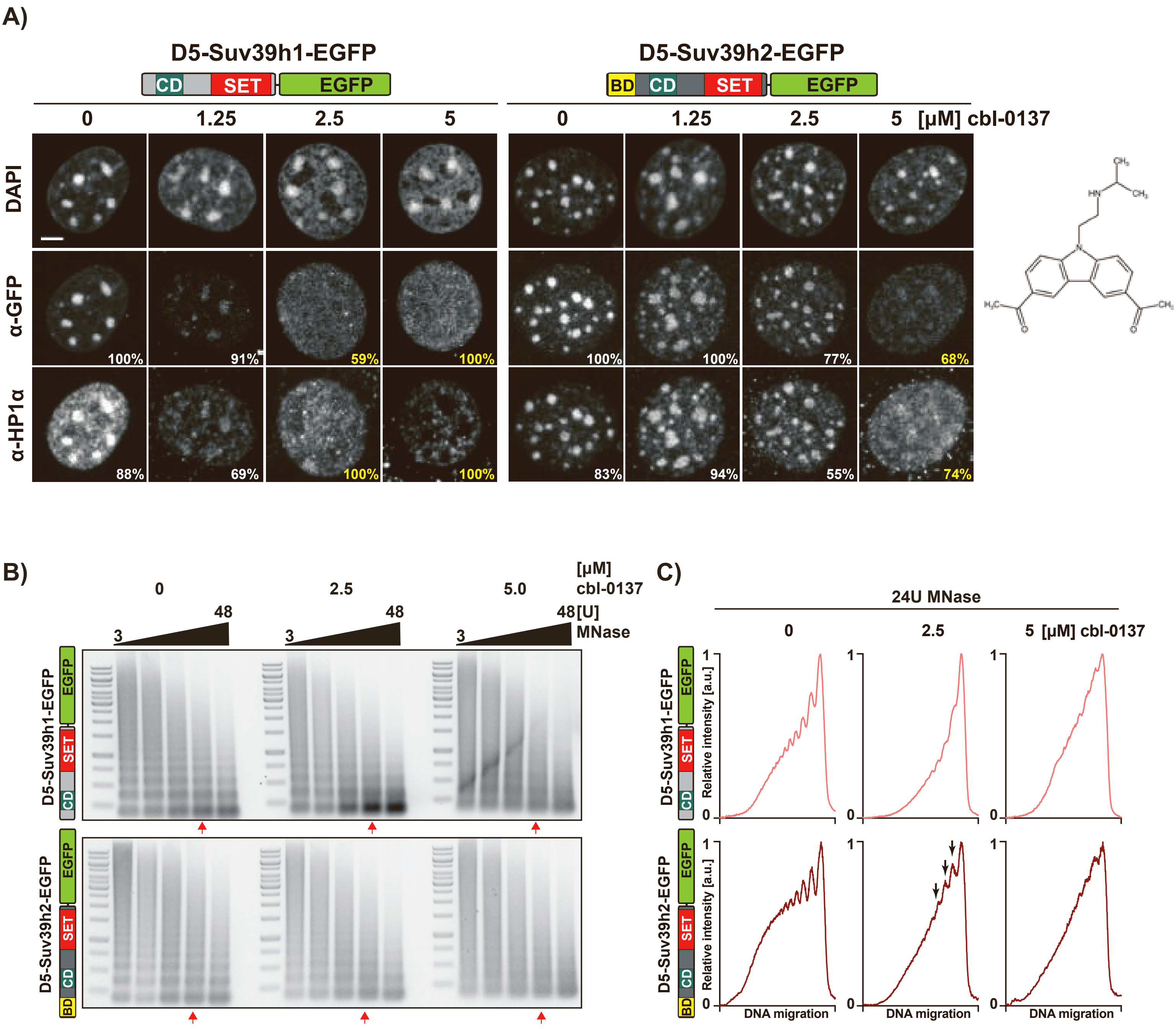

Supplemental figure 7

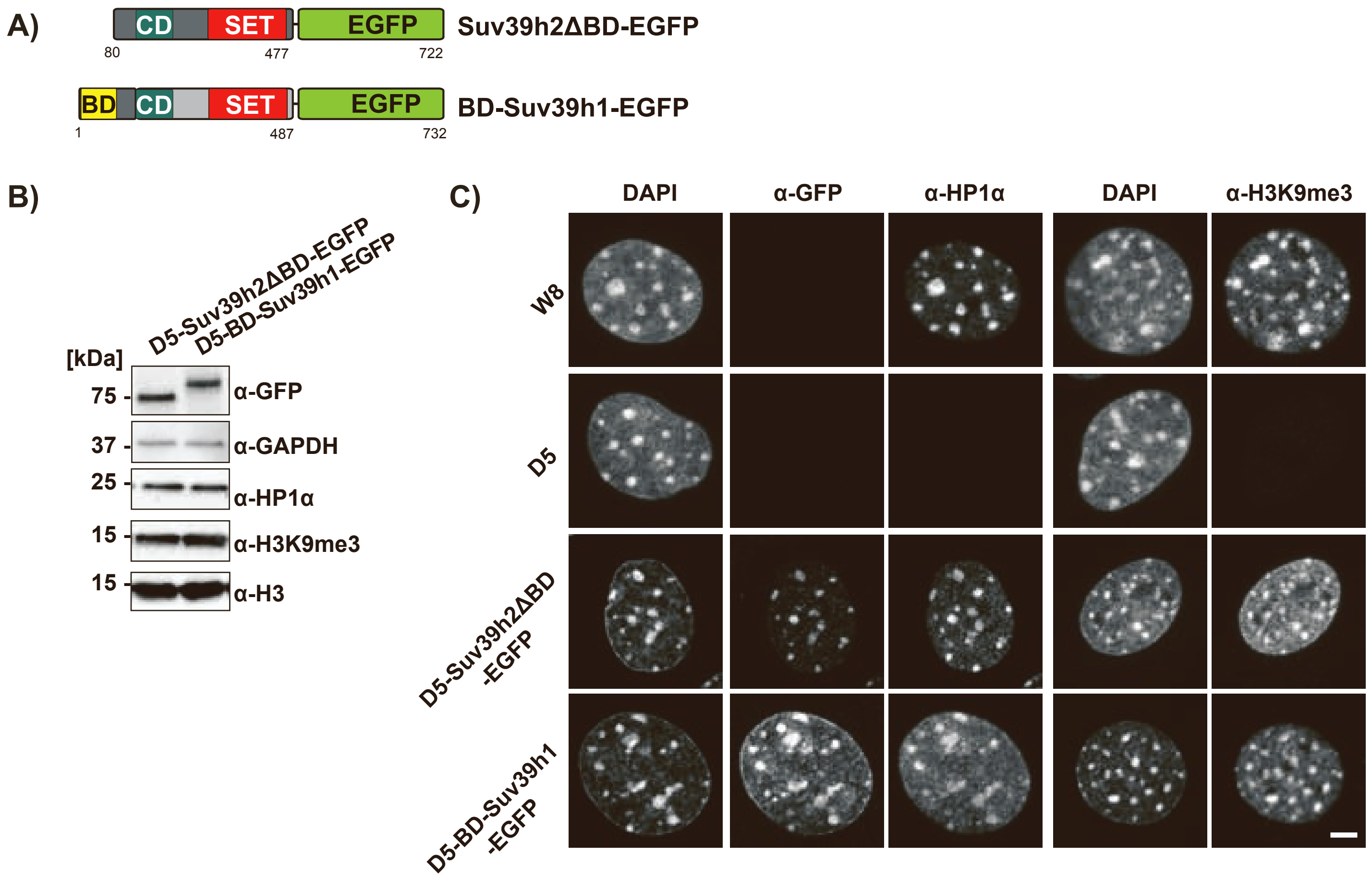

Supplemental figure 8

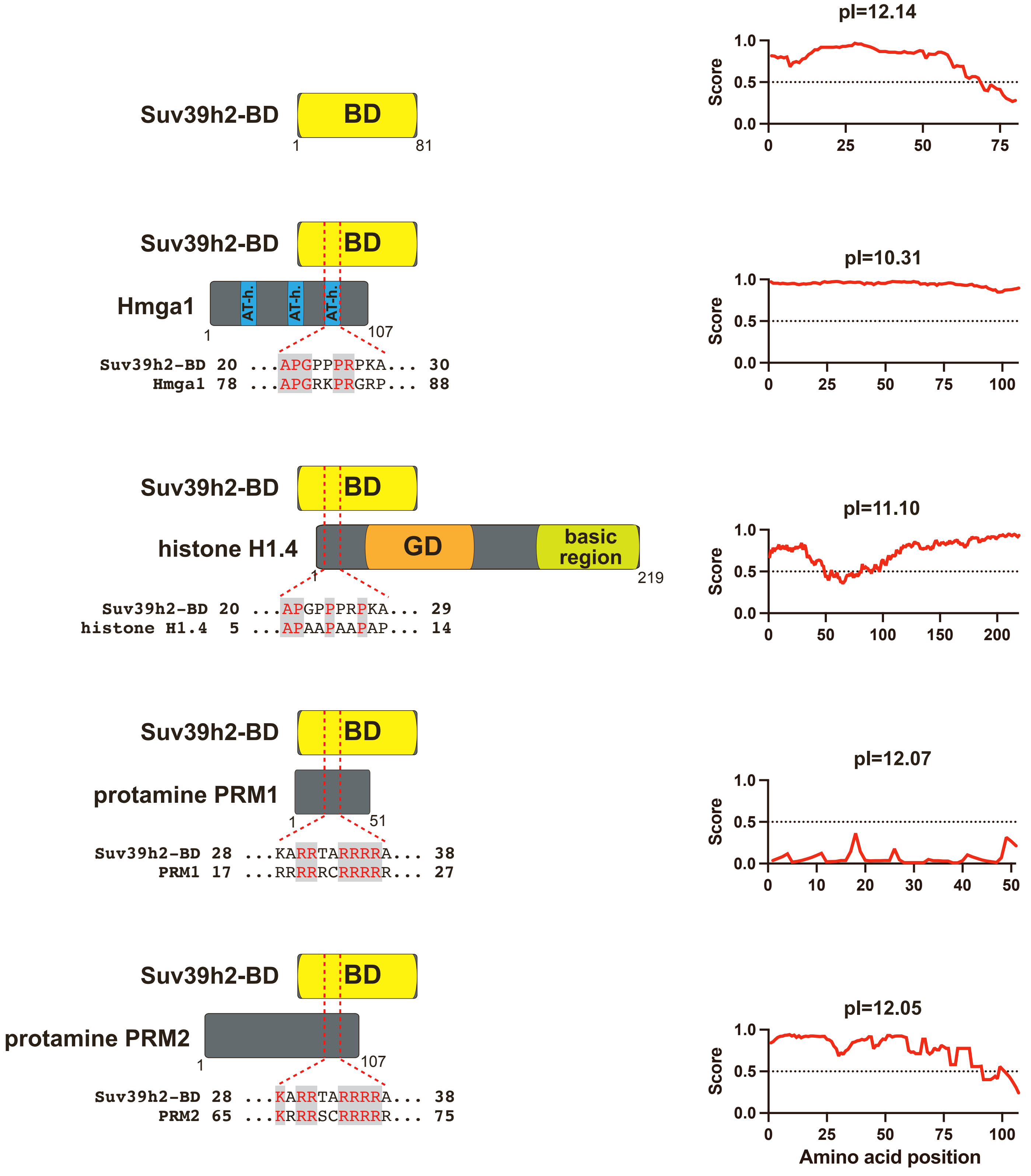

Supplement: Document S1. Figures S1–S8 [file mmc1.pdf]
